# The Awakening Brain is Characterized by a Widespread and Spatiotemporally Heterogeneous Increase in High Frequencies

**DOI:** 10.1002/advs.202409608

**Published:** 2025-03-24

**Authors:** Tamir Avigdor, Guoping Ren, Chifaou Abdallah, François Dubeau, Christophe Grova, Birgit Frauscher

**Affiliations:** ^1^ Analytical Neurophysiology Lab McGill University Montreal QC H3A 2B4 Canada; ^2^ Multimodal Functional Imaging Lab Biomedical Engineering Department McGill University Montreal QC H3A 2B4 Canada; ^3^ Department of Neurology Beijing Tiantan Hospital Capital Medical University Beijing 100070 China; ^4^ China National Clinical Research Center for Neurological Diseases Beijing 100070 China; ^5^ Montreal Neurological Institute and Hospital McGill University Montreal QC H3A 2B4 Canada; ^6^ Multimodal Functional Imaging Lab Department of Physics PERFORM Center/School of Health Concordia University Montreal QC H4B 1R6 Canada; ^7^ Department of Neurology Duke University Medical Center Durham NC 27705 USA; ^8^ Department of Biomedical Engineering Duke Pratt School of Engineering Durham NC 27705 USA

**Keywords:** awakening, connectivity, intracranial EEG, sleep, spectral analysis

## Abstract

Morning awakening is part of everyday life. Surprisingly, information remains scarce on its underlying neurophysiological correlates. Here simultaneous polysomnography and stereo‐electroencephalography recordings from 18 patients are used to assess the spectral and connectivity content of the process of awakening at a local level 15 min before and after the awakening. Awakenings from non‐rapid eye movement sleep are accompanied by a widespread increase in ripple (>80 Hz) power in the fronto‐temporal and parieto‐insular regions, with connectivity showing an almost exclusive increase in the ripple band in the somatomotor, default, dorsal attention, and frontoparietal networks. Awakenings from rapid eye movement sleep are characterized by a widespread and almost exclusive increase in the ripple band in all available brain lobes, and connectivity increases mainly in the low ripple band in the limbic system as well as the default, dorsal attention, somatomotor, and frontoparietal networks.

## Graphical abstract

This work explores the process of morning awakening of the human brain using polysomnography paired with simultaneous stereo‐electroencephalography, a method that provides high spatial and temporal resolution for examining brain activity. Awakening is characterized by an increase in high‐frequency spectral power and connectivity. This suggests that high frequencies play a crucial role in the brain's transition to wakefulness.

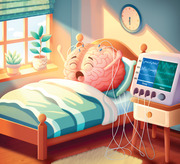

## 1 Introduction

Sleep and wakefulness were classically thought of as a binary phenomenon, i.e., you are either awake or asleep until sleep stages were discovered.^[^ ^1^ ^]^ Awakening in the morning can be viewed as the termination of the sleep process from either rapid‐eye‐movement (REM) or non‐REM (NREM) sleep. Behaviorally, we sometimes feel that it can take some time to be “fully awake”. This is supported by studies that suggest that awakening is a more gradual process^[^ ^2^ ^]^ which can take up to 30 min to return to the wakefulness baseline.^[^ ^3^ ^]^ This has been linked to the incomplete clearance of adenosine at the time of the awakening.^[^ ^4^ ^]^ Evidence from scalp electroencephalography (EEG) showed that awakening, compared to pre‐sleep wakefulness, was characterized by an increase in delta (0.3–4 Hz) and theta (4–8 Hz) activity in parieto‐occipital regions, while the increase in activity in the alpha band (8–12 Hz) was only significant in the eyes closed condition compared to pre‐sleep wakefulness.^[^ ^5^ ^]^ In addition, a decrease in beta (13–30 Hz) activity in the occipital lobe was observed compared to wakefulness.^[^ ^6^ ^]^ The sleep‐wake transition was also shown to have a dependency on the preceding sleep stage.^[^ ^7^ ^]^ However, scalp EEG is spectrally limited by muscle‐related artifacts, especially in the high frequencies over >30 Hz, and more importantly >80 Hz. In addition, scalp EEG has a limited spatial resolution and only provides a global view, making it difficult to perform a careful examination of the local variance in neuronal activity. However, high frequencies may be particularly important in the awakening process as they have been linked to cognition^[^ ^8^ ^]^ and consciousness^[^ ^9^ ^]^ which are regained during this process. Intracranial EEG, a method performed in humans in the context of epilepsy pre‐surgical work‐up^[^ ^10^ ^]^ allows recording from multiple regions in the brain and provides a high‐quality signal with minimal muscle artifacts, a necessity for the study of high frequency activity. More specifically, we used stereo‐EEG (SEEG), a type of intracranial EEG that allows recordings from both superficial and deep structures by inserting depth electrodes into the brain with a higher spatial resolution as opposed to scalp EEG at the location of the implantation probe. The use of sEEG recordings also enables the assessment of high frequencies, providing a movement and muscle‐artefact free signal with high temporal resolution. The use of SEEG recordings in the study of human sleep physiology has enabled the local variability of sleep to be demonstrated between different brain regions and bands.^[^ ^11^, ^12^ ^]^ Interestingly, studies using EEG‐functional magnetic resonance imaging (fMRI) which tried to address the spatial sampling gap have shown that awakening involves a deactivation process that begins in the thalamus and spreads to the cortex.^[^ ^13^, ^14^ ^]^ Additionally, fMRI revealed a reduction in the typical anti‐correlation between regions of networks that are usually anti‐correlated during stabilized wakefulness, specifically between the task‐negative network and the task‐positive networks.^[^ ^15^ ^]^ This reduction also varied depending on the sleep stage.^[^ ^15^ ^]^ Using transcranial Doppler ultrasonography, a reduced cerebral blood flow was observed during NREM compared to REM sleep.^[^ ^15^, ^16^, ^17^ ^]^

Due to these findings, we hypothesize that awakenings will demonstrate heterogeneous spectral and connectivity signatures involving different regions and networks in different bands depending on the prior sleep stage, i.e., awakening from NREM or REM sleep. This is of particular interest as the EEG signature of NREM^[^ ^18^ ^]^ differs from REM^[^ ^19^ ^]^ sleep,^[^ ^20^ ^]^ which suggests a different neural process;^[^ ^21^, ^22^ ^]^ thus awakening from REM compared to NREM might require a different process. Specifically, we are interested in the involvement of high frequency activity, which has been postulated to be important for consciousness for many years,^[^ ^23^, ^24^, ^25^, ^26^, ^27^, ^28^ ^]^ Interestingly however, in patients with epilepsy, loss of consciousness was reported to display the opposite trend.^[^ ^29^ ^]^ Additionally, we propose that the behavioral effects of morning sleep inertia— the grogginess upon waking, that temporarily impairs cognitive performance but fades with time awake^[^ ^30^ ^]^—may reflect variability in the timing and progression of the awakening process. Thus, we were interested in examining the long temporal dynamics both before and after the awakening. Given that sleep and wakefulness can exhibit local changes, it is important to investigate the period leading up to the awakening. Additionally, considering that sleep inertia may imply lingering changes in the minutes following awakening, exploring this time frame is essential for a comprehensive understanding of the whole awakening process. Here we investigated in detail the characteristics of the neural correlates of the process of awakening. We provide the temporal dynamics of local cortical spectral property attributes and network connectivity changes in the high frequency bands with respect to the preceding sleep stage. More specifically, we i) analyzed the amplitude‐sensitive high frequency spectral content and the amplitude‐insensitive phase connectivity content of the final morning awakening from 15 min prior to and 15 min after the awakening, ii) compared these changes to normal wakefulness and stable preceding sleep, and iii) compared these high frequency changes to those of traditional bands in order to obtain a complete picture on how the human brain awakens in the morning.

## 2 Results

**Figure 1:**
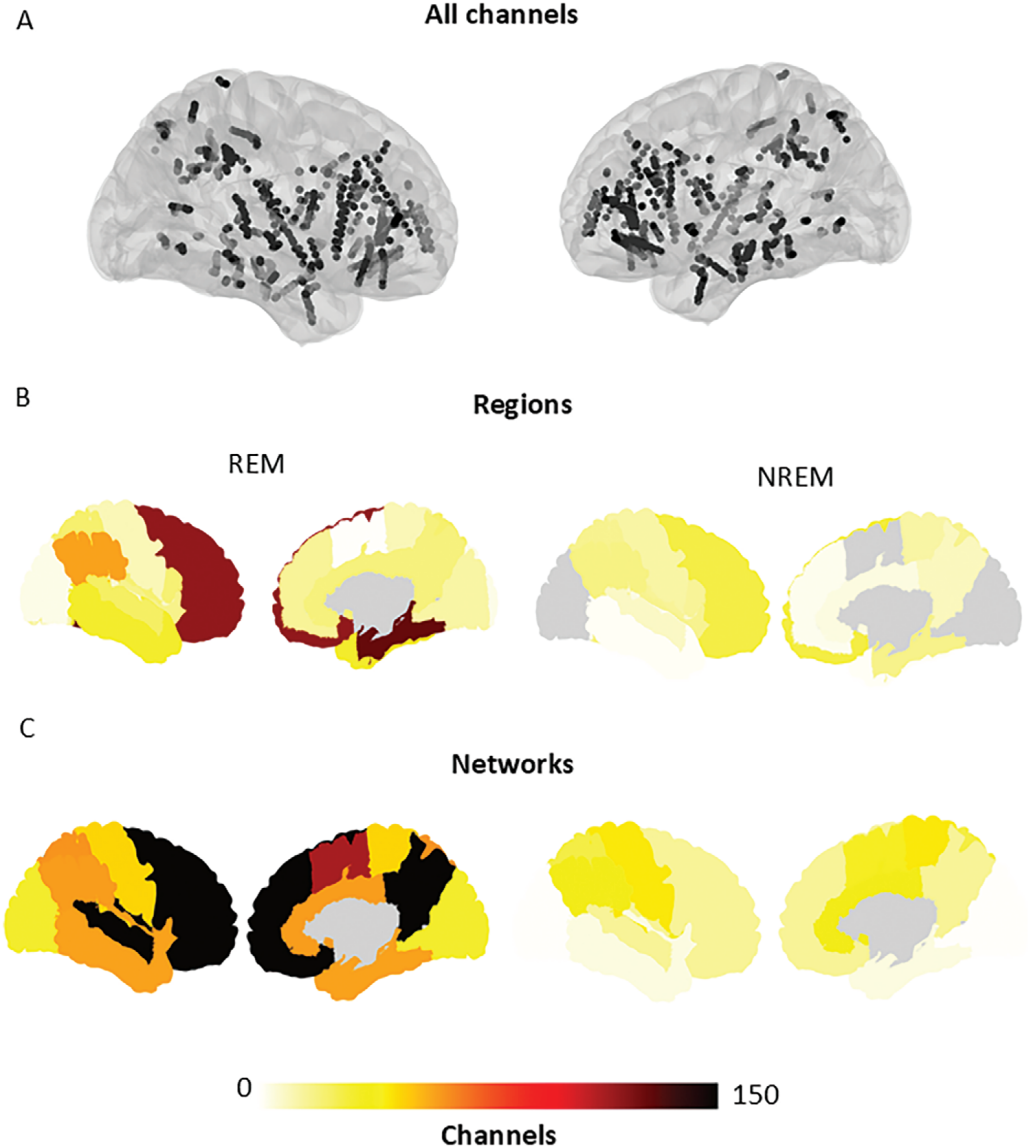
Available coverage in the regional and network atlases. A) All available healthy channels from all patients. B) Regional coverage in the MICCAI 17 anatomical atlas. C) Coverage of the networks in the Yeo 7 atlas. The color gradient indicates the number of channels across all patients in each region.

We analyzed the morning awakenings of 18 patients with drug‐resistant focal epilepsy who underwent simultaneous polysomnography (PSG) and SEEG electrode implantations as part of their pre‐surgical epilepsy evaluation. We assessed the local changes in power and connectivity that occur during the awakening process in various brain regions and networks. We examined all SEEG channels deemed healthy by an epileptologist, as well as those determined to be in the gray matter, totaling 695 channels (38.6 ± 33.4 per patient). We focus on high‐frequency bands but test all bands to evaluate the specificity of the findings, for every region or for every network (**Figure** **1**; Figures S1, S2; and Table S1, Supporting Information) with sufficient coverage (≥3 patients and 5 channels). We report data from 7 patient awakenings from NREM sleep and 11 from REM sleep. Finally, we report results for 6 regions and 5 networks for NREM awakenings, and 14 regions and 7 networks for REM awakenings (Figure 1; Figures S1, S2, and Table S1, Supporting Information).

**Figure 2:**
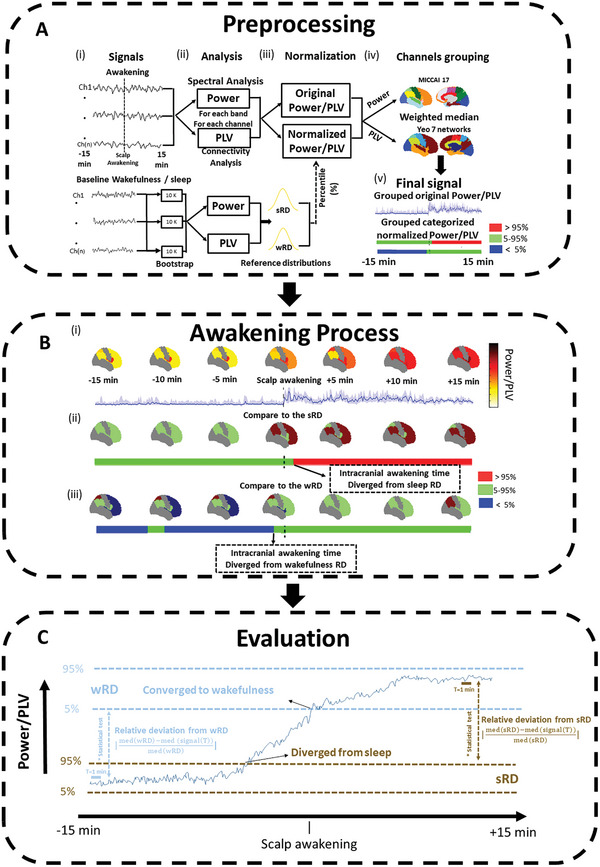
The overall scheme of the analysis process. A) Preprocessing involves several steps. i) The awakening signals from 15 min before to 15 min after the scalp awakening (SA) were taken, as well as baseline segments from the previous sleep from −15 to −10 min before the SA, and wakefulness from the prior day. These baseline segments were then furthered bootstrapped. ii) Analysis was performed in each frequency band to assess the spectral properties using Morlet wavelet, this was done for each contact, and then all the channels were grouped using the MICCAI 17 atlas. In addition, connectivity was estimated using phase locking value (PLV) for each available channel pair within the same network, or between two different networks available, in the Yeo 7 networks atlas. A similar analysis (power and PLV) was also applied to every bootstrapped baseline segment, therefore creating two normative reference distributions (RD), one from the previous sleep (sRD) and one from wakefulness (wRD). iii) Then, in addition to the original power/PLV, we also estimated their corresponding percentiles in comparison with the sRD and the wRD. iv) The original power/PLV and the percentile signals were combined using a weighted median for all channels and patients within a given region or network. v) The final signals for analysis are presented. The original power/PLV signal and two categorized percentiles signals one with respect to the sRD and one with respect to the wRD. The categorized percentiles are labelled as above 95% (red) within 5–95% (green) and below 5% (blue). B) Description of the awakening process of the i) power or PLV every 5 min for each available region/network, as well as an example of the continuous temporal changes in one region or network (in this example the frontal lobe) with a shaded interquartile in the region. ii) The categorized percentile with respect to the sRD and iii) the categorized percentile with respect to the wRD. An example of the corresponding continuous changes in the representative lobe is also shown. The points where the percentile diverges from the sRD and converges to the wRD are marked as the intracranial awakening (IA) points. C) Evaluation of the awakening was done from (1) the time before the power/PLV converges to wRD and (2) after it diverges from the sRD, marked by the IA time points, i.e., the wakefulness IA and the sleep IA. A channel level analysis was conducted using a Wilcoxon rank sum test with a Benjamini‐Hochberg false discovery rate correction. Then, we calculated the relative deviation from the RD as the weighted median (WM) of the percentage difference at the channel level between the median awakening signal to the median RDs.

All analysis is performed at the channel level to mitigate any confounding factors between patients or regional differences. First, the time of scalp awakening (SA) is determined visually by a board‐certified neurophysiologist for each patient using scalp EEG. In the second step, the signal is analyzed 15 min prior to and 15 min after that time (**Figure** **2**). We assessed this period and compared it to reference distributions (RD) of full wakefulness (wRD) and prior sleep (sRD). We are interested in determining when each region or network “wakes up”, i.e., if there is local sleep or wakefulness that might be different from the global state and if so when it changes. For this purpose, we assess when the signal diverges out of the bounds of sleep and when it converges back to the bounds of wakefulness (see Experimental Section). Thus, we aim to identify a time point, which we named intracranial awakening (IA) times, for each region or network from which we can safely say that the signal is no longer within the bounds of sleep, and a time point where it will be back within the bounds of wakefulness. After having identified these time points, we assess segments prior to the convergence back to wakefulness and segments after the divergence from sleep. We assess them to further validate the statistical differences using the Wilcoxon rank sum test with a Benjamini‐Hochberg correction, effect sizes using Cliff's Delta, and overall magnitude differences between these periods compared to sleep or wakefulness using a relative deviation from the RD (see Experimental Section). This is done to not only give an estimated time of local awakening but also to assess the level of change during this process. The final morning awakening spectral content was estimated using Welch's method and phase connectivity properties using the phase locking value (PLV). This was done for each patient, spanning from 15 min before to 15 min after the awakening (Figure 2). Results reported below are for bands, regions, and networks that displayed significant changes (**Tables** **1**, **2**, **3**, **4**, for a full description of the results for all the bands, regions, and networks please refer to the Supporting information (Tables S2–S5, Supporting Information). IA times in relation to the wakefulness baseline remained largely (90%) identical when the wRD was constructed using segments from the late afternoon or the morning, and were all between 5–10 s (Tables S2–S5, Supporting Information). Spurious spikes detections accounted for <1% of the signal, and the results did not change with or without the removal of spurious spikes.

**Table 1:** Spectral density results of awakening from NREM sleep. Reported here are spectral results for all bands which displayed a significant difference between the awakening process to the wakefulness or sleep reference distributions (wRD and sRD respectively). The results are presented as the time in seconds from the intracranial awakening (IA) when compared to the RDs. Times are given for the convergence to the wRD and the divergence from the sRD. The difference between the awakening process and the RD prior to convergence or after the divergence was tested on the channel level for all channels in the region using a paired Wilcoxon test and assessed with Cliff's d. The magnitude of the difference between the awakening process to the RDs is reported as relative deviation, which is the change in percentage compared to the RDs. The direction of change is represented as ↑↓ if the time after the awakening corresponded to an increase ↑ or decrease ↓ when compared to the reference distribution. Note: 1 only significant bands and regions are reported (*p* < 0.05 after correction for performing of multiple comparisons).

Region	NREM			
	Relative deviation from wRD		Relative deviation sRD	
Inferior parietal lobule	delta	−30s (↑ 77–81%; d = 0.77‐86; *p* < 0.001)	delta	45s (↓95–270%; d = 0.59–0.80; *p* < 0.001)
			theta	350s (↓ 77–83Y%; d = 0.23–0.33; *p* < 0.01)
			low gamma	50s (↑ 24–33%; d = 0.19–0.31; *p* < 0.01)
	theta	−40s (↑ 55–61%; d = 0.47–0.49; *p* < 0.001)	low ripple	200s (↑ 35–47%; d = 0.25–0.35; *p* < 0.01)
			high ripple	150s (↑ 23–44%; d = 0.26–0.66; *p* < 0.001)
Central operculum and opercular part of inferior frontal gyrus	delta	−65s (↓ 30–63%; d = 0.35–0.45; *p* < 0.001)	delta	215s (↑ 124–165%; d = 0.46–0.53; *p* < 0.01)
	high ripple	−40s (↓ 55–60%; d = 0.05–0.08; *p* < 0.01)	low ripple	230s (↑ 13–22%; d = 0.03–0.26; *p* < 0.001)
			high ripple	235s (↑ 14–34%; d = 0.12–0.16; *p* < 0.01)
Superior, middle, and orbital frontal gyri and anterior part of inferior frontal gyrus	delta	−30s (↓ 40–46%; d = 0.39–0.48; *p* < 0.001)	delta	45s (↓ 118–165%; d = 0.38–0.55; *p* < 0.001)
			Beta	30s (↑ 11–25%; d = 0.03‐0.13; *p* < 0.01)
	beta	−45s (↓ 39–59%; d = 0.16‐24; *p* < 0.001)	low gamma	65s (↑ 9–13%; d = 0.04–0.09; *p* < 0.01)
			high gamma	230s (↑ 9–19%; d = 0.08–0.09; *p* < 0.001)
	high ripple	−40s (↓ 62–69%; d = 0.38–0.52; *p* < 0.001)	low ripple	190s (↑ 15–39%; d = 0.14–0.23; *p* < 0.01)
			high ripple	50s (↑ 17–30%; d = 0.38–0.49; *p* < 0.001)
Insula	high ripple	−40s (↓ 80–90%; d = 0.11–0.13; *p* < 0.01)	delta	40s (↓ 77–105%; d = 0.16–0.24; *p* < 0.01)
			high ripple	230s (↑ 27–33%; d = 0.02–0.13; *p* < 0.01)
Superior parietal lobule	high ripple	−215s (↑ 21–51%; d = 0.34–0.74; *p* < 0.001)	delta	45s (↓ 56–161%; d = 0.53–0.55; *p* < 0.001)
			high ripples	50s (↑ 23–44%; d = 0.09–0.66; *p* < 0.001)
Middle and inferior temporal gyrus, temporal pole, and planum polare	high ripple	−235s (↓ 21–24%; d = 0.24–0.48; *p* < 0.001)	delta	20s (↓ 44–69%; d = 0.42–0.53; *p* < 0.001)
			low gamma	50s (↑ 6–14%; d = 0.14–0.18; *p* < 0.001)
			low ripple	50s (↑ 18–28%; d = 0.11–0.26; *p* < 0.01)
			high ripple	50s (↑ 19–24%; d = 0.06–0.23; *p* < 0.01)

### 2.1 Awakenings from NREM Sleep are Associated with Widespread Power Changes

**Figure 3:**
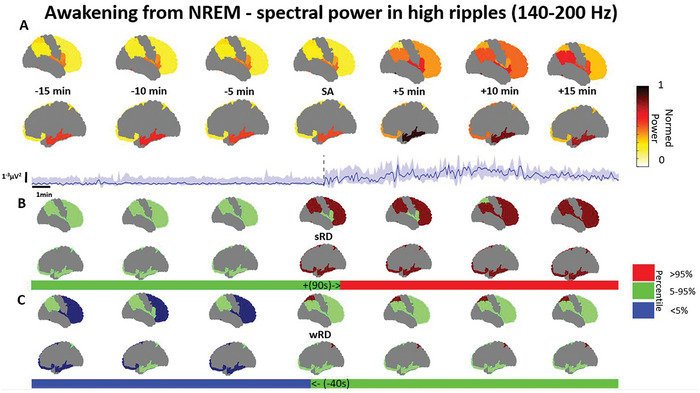
Spectral power in the high ripple band increases upon awakening from NREM sleep. A) Power from every available region across the brain is shown from 15 min before to 15 min after the scalp awakening (SA), along with a regional example of the power with a quartile range as the shaded area, and the respective intracranial awakening times for the sleep reference distribution (sRD) and the wakefulness RD (wRD). B) The overall percentile power in relation to the sRD is presented for every region along with the same regional example as a bar. C) The overall percentile power in relation to the wRD is presented for every region with the same regional example as a bar. Note: Power represents the weighted median power in each region, normalized across the entire brain. The reference distribution percentiles are based on the weighted median in each region, which are then categorized as >95%, within 5–95%, or < 5%. Continuous power examples and comparisons to wakefulness and sleep reference distributions are illustrated for the superior, middle, and inferior frontal regions. Deviations < 5% or >95% percentiles are deemed significant. Regions included in the example are the superior, middle, and orbital frontal gyri, the anterior part of the inferior frontal gyrus, the insula, the central operculum, the opercular part of the inferior frontal gyrus, the superior parietal lobule, the middle and inferior temporal gyrus, temporal pole, planum polare, and the inferior parietal lobule. Data was presented for 6 patients from a total of 118 channels, ranging between 15–32 per region (Full description in Table S1A, Supporting Information).

Awakening from NREM sleep was associated with spectral changes in multiple bands and regions, with heterogeneous IA times compared to the SA (Table 1). Awakening from NREM displayed a power increase in the high ripple band in the superior, middle, and orbital frontal gyri and anterior part of the inferior frontal gyrus, the insula, the central operculum, the opercular part of inferior frontal gyrus, the superior parietal lobule, the middle and inferior temporal gyrus, temporal pole, planum polare, and the inferior parietal lobule (**Figure** **3**). The relative deviation from sRD ranged between 14–44% (d = 0.02–0.66; *p* < 0.01) and started between 50 to 235 s after the SA. The relative deviation from wRD ranged between 21–90% (d = 0.05–0.74; *p* < 0.01) in the high ripple band and terminated between 235 to 40 s before the SA.

As expected, lower frequencies in the delta band (Figure S3, Supporting Information) displayed a widespread decrease during the awakening process, involving all regions. The relative deviation from sRD ranged between 44–270% (d = 0.16–0.80; *p* < 0.001) and started between 20 to 215 s after the SA. The relative deviation from wRD ranged between 30–81% (d = 0.35–0.86; *p* < 0.001) terminating between 30 to 65 s before the SA. In addition, a theta and alpha decrease, and a beta increase were observed mainly in frontal regions during the awakening process (Table 1). The awakening process showed a correlation between the power of scalp EEG at positions Fz or Cz and intracranial EEG, primarily in the frontal neocortex at low frequencies, during awakening from NREM sleep. As expected, there was no correlation with remote regions or structures situated deep in the brain (Tables S7 and S8, Supporting Information).

### 2.2 High Frequency Phase Connectivity Increased in the Multiple Networks During the Awakening from NREM Sleep

**Figure 4:**
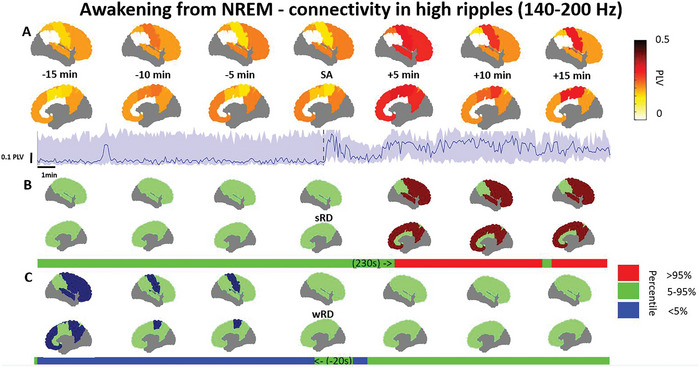
Phase connectivity within networks in the high ripple band increases with awakening from NREM. A) Phase locking value (PLV) within each available network in the brain is shown from 15 min before to 15 min after the scalp awakening (SA), along with a network example from every available network. Quartile ranges are represented by the shaded area. The respective intracranial awakening times for sleep reference distribution (sRD) and wakefulness RD (wRD). B) The overall PLV percentile in relation to the sRD within every network is complemented by a network example in the bar. C) The overall PLV percentile in relation to the wRD is presented by the bar, with an accompanying network example above. Note: PLV represents the weighted median power within each network. The reference distribution percentiles are based on the weighted median within each network, which are then categorized as >95%, within 5–95%, or < 5%. Continuous PLV examples and comparisons to wakefulness and sleep reference distributions are illustrated for the somatomotor network. Deviations below the 5% or above the 95% percentiles are deemed significant. Included networks are: somatomotor, dorsal attention, frontoparietal, and the default mode network. Data was presented for 6 patients from a total of 178 channels, ranging between 5–43 per network (Full description in Table S1B, Supporting Information).

We then further explored and assessed the phase connectivity content of the awakening from NREM sleep within and between all available networks of the Yeo 7 network atlas. Connectivity‐wise, awakening from NREM showed maximal changes occurring in only one or two frequency bands, mostly within the ripple bands (Table 2). Connectivity increased during the awakening from NREM in the high ripple band within the somatomotor, default, dorsal attention, and frontoparietal networks (**Figure** **4**). The relative deviation from sRD ranged between 20–61% (d = 0.15–0.79; *p* < 0.001) and started between 45 to 230 s after the SA. The relative deviation from wRD within the somatomotor and dorsal attention networks ranged between 29–124% (d = 0.79–0.85; *p* < 0.001), and terminated 20 to 345 s prior to the SA. Furthermore, connectivity increased in the ripple bands between the following networks: somatomotor and ventral attention network, frontoparietal and default mode network, dorsal attention and both the frontoparietal network and default mode network, and both the ventral attention and frontoparietal networks. The relative deviation from sRD ranged between 12–59% (d = 0.13–0.78; *p* < 0.01) and started between 45 to 390 s after the SA. The relative deviation from wRD ranged between 125–288% (d = 0.10 –0.72; *p* < 0.001) and terminated 20 to 40 s prior to the SA, but only between the somatomotor and ventral attention network, somatomotor network, and default mode network, as well as between the default mode network and ventral attention network. Lastly, the connectivity of the beta bands showed a minor decrease between the dorsal attention network and both the frontoparietal and default mode network, and between the default mode network and frontoparietal network. The relative deviation from sRD ranged between 3–27% (d = 0.07–0.33; *p* < 0.001) and started between 95 s prior to the SA to 110 s after the SA. Unexpectedly, no divergence from the wRD was observed during the awakening process. Interestingly no significant changes in the PLV connectivity were observed in any other frequencies.

**Table 2:** Phase connectivity results of awakening from NREM. Reported here are phase locking value (PLV) results for all bands which displayed a significant difference between the awakening process to the wakefulness or sleep reference distributions (wRD and sRD respectively). The results are presented as the time in seconds from the intracranial awakening (IA) when compared to the RDs. Times are given for the convergence to the wRD and the divergence from the sRD. The difference between the awakening process and the RD prior to convergence or after the divergence was tested on the channel level for all channels‐pairs within a network or between two different networks, using a paired Wilcoxon test and assessed with Cliff's d. The magnitude of the difference between the awakening process to the RDs is reported as relative deviation, which is the change in percentage compared to the RDs. The direction of change is represented as ↑↓ if the time after the awakening corresponded to an increase ↑ or decrease ↓ when compared to the reference distribution. ND‐ never diverged from the RD in any frequency band. Note: only significant bands and regions are reported (*p* < 0.05 after correction for performing of multiple comparisons).

Network	NREM			
	Relative deviation from wRD		Relative deviation from sRD	
Somatomotor	high ripple	−20s (↓ 119–124%; d = 0.83–0.85; *p* < 0.001)	high ripple	230s (↑ 20–29%; d = 0.15–0.78; *p* < 0.001)
Somatomotor ‐Ventral attention	high ripple	−40s (↓ 153–188%; d = 0.65–0.68; *p* < 0.001)	high ripple	255s (↑ 20–28%; d = 0.25–0.82; *p* < 0.001)
Somatomotor ‐Frontoparietal	ND		high ripple	370s (↑ 20–44%; d = 0.20–0.66; *p* < 0.001)
Somatomotor – Default	high ripple	−20s (↓ 229–288%; d = 0.70–0.72; *p* < 0.001)	high ripple	390s (↑ 16–35%; d = 0.35–0.76; *p* < 0.001)
Dorsal attention	high ripple	−345s (↓ 29–56%; d = 0.79–0.83; *p* < 0.001)	low ripple	50s (↑ 18–32%; d = 0.13–0.32; *p* < 0.001)
			high ripple	95s (↑ 48–61%; d = 0.34–0.61; *p* < 0.001)
Dorsal attention – Frontoparietal	ND		beta	110s (↓ 9–11%; d = 0.17–0.30; *p* < 0.001)
			low ripple	80s (↑ 25–29%; d = 0.41–0.46; *p* < 0.001)
			high ripple	45s (↑ 49–51%; d = 0.29–0.56; *p* < 0.001)
Dorsal attention – Default	ND		beta	−95s (↓ 8–27%; d = 0.13–0.33; *p* < 0.001)
			low ripple	50s (↑ 18–23%; d = 0.26–0.39; *p* < 0.01)
Default	ND		low ripple	220s (↑ 12–17%; d = 0.15–0.17; *p* < 0.001)
			high ripple	200s (↑ 50–59%; d = 0.23–0.35; *p* < 0.001)
Default – Ventral attention	high ripple	−40s (↓ 125–147%; d = 0.10–0.22; *p* < 0.01)	low ripple	350s (↑ 12–24%; d = 0.20–0.22; *p* < 0.01)
			high ripple	240s (↑ 18–22%; d = 0.11–0.16; *p* < 0.001)
Default – Frontoparietal	ND		beta	55s (↓ 3–20%; d = 0.07–0.13; *p* < 0.001)
			high ripple	380s (↑ 13–18%; d = 0.53–0.54; *p* < 0.001)
Frontoparietal	ND		high ripple	45s (↑ 34–40%; d = 0.77–0.79; *p* < 0.001)

### 2.3 Awakening from REM Sleep is Associated with a Widespread Power Increase Exclusively in High Frequencies

The observation that high frequencies are associated with the awakening from NREM sleep raised the question of whether they may also be involved in awakenings from REM sleep. REM sleep, unlike NREM sleep, is classically characterized by a “wake‐like” EEG signal. However, high frequencies are difficult to detect on the scalp. In contrast, SEEG offers a unique opportunity to investigate local changes in high frequencies. Therefore, we also explored the process of awakening from REM sleep.

**Figure 5:**
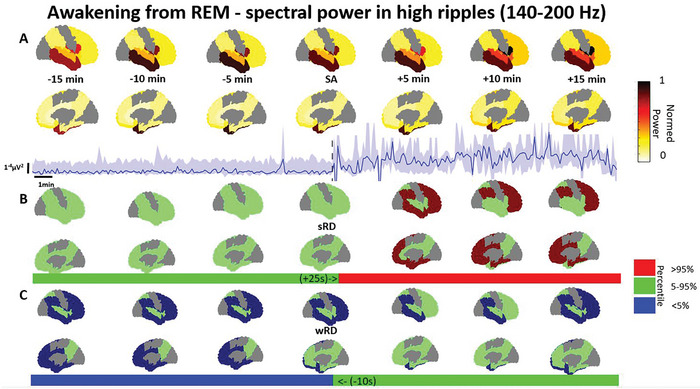
Spectral power in the high ripple band increases upon awakening from REM sleep. A) Power from every available region across the brain is shown from 15 min before to 15 min after the scalp awakening (SA), along with a regional example of the power with a quartile range as the shaded area, and the respective intracranial awakening times for sleep reference distribution (sRD) and wakefulness RD (wRD). B) The overall percentile power in relation to the sRD is presented for every brain region, complemented by the same regional example as a bar. C) The overall percentile power in relation to the wRD is presented for every brain region with the same regional example as a bar. Note: Power represents the weighted median power in each region, normalized across the entire brain. The reference distribution percentiles are based on the weighted median in each region, which are then categorized as over 95%, within 5–95%, or below 5%. Continuous power examples and comparisons to wakefulness and sleep reference distributions are illustrated for the middle and inferior temporal gyrus, temporal pole, and planum polare regions. Deviations below the 5% or above the 95% percentiles are deemed significant. Included regions are: medial occipital lobe, medial and basal temporal region, medial parietal lobe, superior parietal lobule, superior temporal gyrus, medial frontal cortex (including medial segment of superior frontal gyrus), inferior parietal lobule, superior, middle, and orbital frontal gyri and anterior part of inferior frontal gyrus, insula, middle and inferior temporal gyrus, temporal pole, and planum polare, anterior and middle cingulate gyrus, frontal operculum, medial and basal temporal region, transverse temporal gyrus and planum temporale. Data was presented for 12 patients from a total of 525 channels, ranging between 8–127 per region (Full description in Table S1A, Supporting Information).

Awakening from REM sleep was associated with a widespread power increase almost exclusively in the low and high ripple bands (Table 3). These increases were noted to occur widespread over the brain (**Figure** **5**, full list in Table 3). The relative deviation from sRD ranged between 5–39% (d = 0.03–0.44; *p* < 0.01) and started between 50 s prior to the SA to 260 s after the SA. The relative deviation from wRD ranged between 36–88% (d = 0.02–0.57; *p* < 0.01) and terminated 45 s before to 45 s after the SA, with some regions failing to converge back. Interestingly, changes in lower frequencies (delta, alpha, and beta) were minimal in most regions, except in the inferior parietal lobule, medial and basal temporal region, middle and inferior temporal gyrus, temporal pole, planum polare, superior, middle, and orbital frontal gyri, anterior part of inferior frontal gyrus, and the superior temporal gyrus. This manifested as a relative deviation from sRD ranging between 11–87% (d = 0.11–73; *p* < 0.01) starting between 480 s before to 50 s after the SA, but never diverging from the wRD.

**Table 3:** Spectral density results of awakening from REM sleep. Reported here are spectral results for all bands which displayed a significant difference between the awakening process to the wakefulness or sleep reference distributions (wRD and sRD respectively). The results are presented as the time in seconds from the intracranial awakening (IA) when compared to the RDs. Times are given for the convergence to the wRD and the divergence from the sRD. The difference between the awakening process and the RD prior to convergence or after the divergence was tested on the channel level for all channels in the region using a paired Wilcoxon test and assessed with Cliff's d. The magnitude of the difference between the awakening process to the RDs is reported as relative deviation, which is the change in percentage compared to the RDs. The direction of change is represented as ↑↓ if the time after the awakening corresponded to an increase ↑ or decrease ↓ when compared to the reference distribution. ND‐ never diverged from the RD in any frequency band, NC‐ never converged back the RD. Note that while the medial and basal temporal region never significantly converged to the wRD they did show an upward trend. Note: only significant bands and regions are reported (*p* < 0.05 after correction for performing of multiple comparisons).

Region	REM			
	wRD		sRD	
Anterior and middle cingulate gyrus	low ripple	−45s (↓ 41–47%; d = 0.31–0.33; *p* < 0.01)	low ripple	260s (↑ 5–21%; d = 0.21–0.44; *p* <.01)
	high ripple	−40s (↓ 36–41%; d = 0.38–0.42; *p* < 0.01)	high ripple	45s (↑ 5–22%; d = 0.22–0.29; *p* < 0.01)
Central operculum and opercular part of inferior frontal gyrus	high ripple	−35s (↓ 59–62%; d = 0.16–0.22; *p* < 0.01)	ND	
Inferior parietal lobule	low ripple	45s (↓ 42–47%; d = 0.11–0.27; *p* < 0.001)	delta	−350s (↓ 13–87%; d = 0.11–0.73; *p* < 0.001)
	high ripple	NC (↓ 40–65%; d = 0.02–0.28; *p* < 0.05)	low ripple	165s (↑ 12–15%; d = 0.03–0.15; *p* < 0.01)
Insula	high ripple	ND	high ripple	305s (↑ 5–17%; d = 0.17–0.25; *p* < 0.05)
Medial and basal temporal region	high gamma	NC (↓ 52–92%; d = 0.31–0.34; *p* < 0.001)	delta	−480s (↓ 13–72%; d = 0.30–0.42; *p* < 0.001)
	low ripple	NC (↓ 41–85%; d = 0.15–0.52; *p* < 0.001)	low ripple	55s (↑ 13–19%; d = 0.30–0.31; *p* < 0.001)
	high ripple	NC (↓ 49–88%; d = 0.35–0.57; *p* < 0.01)		
Medial frontal cortex	low ripple	45s (↓ 37–40%; d = 0.18–0.19; *p* < 0.001)	low ripple	35s (↑ 6–25%; d = 0.18–0.30; *p* < 0.01)
	high ripple	−40s (↓ 36–43%; d = 0.18–0.19; *p* < 0.001)	high ripple	45s (↑ 12–26%; d = 0.09–0.12; *p* < 0.001)
Middle and inferior temporal gyrus, temporal pole, and planum polare	high gamma	−5s (↓ 46–50%; d = 0.22–0.23; *p* < 0.001)	delta	−405s (↓ 19–58%; d = 0.23–0.43; *p* < 0.001)
			beta	25s (↑ 15–19%; d = 0.15–0.20; *p* < 0.001)
	low ripple	10s (↓ 81–86%; d = 0.47–0.48; *p* < 0.001)	high gamma	75s (↑ 9–13%; d = 0.04–0.15; *p* <.001)
	high ripple	−10s (↓ 54–77%; d = 0.35–0.36; *p* < 0.001)	low ripple	−50s (↑ 18–24%; d = 0.20–0.44; *p* < 0.001)
			high ripple	−25s (↑ 22–27%; d = 0.24–0.34; *p* < 0.001)
Superior, middle, and orbital frontal gyri and anterior part of inferior frontal gyrus	low ripple	0s (↓ 39–44%; d = 0.16–0.19; *p* < 0.001)	delta	−480s (↓ 37–87%; d = 0.16–0.57; *p* < 0.01)
	high ripple	0s (↓ 39–42%; d = 0.12–0.14; *p* < 0.001)	low ripple	55s (↑ 10–29%; d = 0.11–0.36; *p* < 0.001)
			high ripple	50s (↑ 14–31%; d = 0.08–0.20; *p* < 0.001)
Superior temporal gyrus	low ripple	−30s (↓ 43–47%; d = 0.25–0.27; *p* < 0.001)	alpha	50s (↑ 23–40%; d = 0.14–0.42; *p* < 0.001)
			beta	−40s (↑ 11–24%; d = 0.11–0.24; *p* < 0.001)
	high ripple	ND	low ripple	55s (↑ 19–36%; d = 0.21–0.44; *p* < 0.01)
			high ripple	−20s (↑ 12–39%; d = 0.09–0.27; *p* < 0.001)

### 2.4 Connectivity Increases in the Ripple Bands within the Default Mode, Dorsal Attention, Somatomotor, and Frontoparietal networks During the Awakening from REM Sleep

We further investigated the phase connectivity content of the awakening from REM sleep within and between all available networks in the Yeo 7 network atlas. This investigation revealed that awakenings from REM were associated almost exclusively with increases in the low ripple band across multiple networks (Table 4).

**Table 4:** Phase connectivity results of awakening from REM. Reported here are phase locking value (PLV) results for all bands which displayed a significant difference between the awakening process to the wakefulness or sleep reference distributions (wRD and sRD respectively). The results are presented as the time in seconds from the intracranial awakening (IA) when compared to the RDs. Times are given for the convergence to the wRD and the divergence from the sRD. The difference between the awakening process and the RD prior to convergence or after the divergence was tested, on the channel level for all channels‐pairs within a network or between two different networks, using a paired Wilcoxon test and assessed with Cliff's d. The magnitude of the difference between the awakening process to the RDs is reported as relative deviation, which is the change in percentage compared to the RDs. The direction of change is represented as ↑↓ if the time after the awakening corresponded to an increase ↑ or decrease ↓ when compared to the reference distribution. ND‐ never diverged from the RD in any frequency band. NC‐ never converged back to the RD. Note: only significant bands and regions are reported (*p* < 0.05 after correction for performing of multiple comparisons).

Network	REM			
	wRD		sRD	
Limbic	low ripples	−40s (↓ 36–40%; d = 0.30–0.38; *p* < 0.001)	low ripples	50s (↑ 28–39%; d = 0.30–0.35; *p* < 0.001)
Limbic – Ventral attention	ND		low ripples	50s (↑ 21–24%; d = 0.34–0.44; *p* < 0.001)
Limbic – Visual	high ripples	NC (↑ 21–24%; d = 0.21–0.35; *p* < 0.001)	high ripples	230s (↓ 6–7%; d = 0.20–0.23; *p* < 0.01)
Limbic – Somatomotor	high ripples	−455s (↑ 15–16%; d = 0.51‐54; *p* < 0.001)	low ripples	45s (↑ 22–23%; d = 0.31–0.41; *p* < 0.001)
Somatomotor – Ventral attention	high ripples	−75s (↑ 14–42%; d = 0.62–0.65; *p* < 0.001)	low ripples	45s (↑ 21–22%; d = 0.14–0.38; *p* < 0.01)
Limbic – Visual	high ripples	NC (↑ 21–41%; d = 0.21–0.55; *p* < 0.001)	low ripples	95s (↑ 25–26%; d = 41‐47; *p* < 0.001)
Limbic – Default	ND		low ripples	45s (↑ 20–24%; d = 0.35–0.41; *p* < 0.001)
			high ripples	390s (↑ 14–19%; d = 0.02–0.29; *p* < 0.001)
Limbic – Frontoparietal	ND		low ripples	45s (↑ 28–39%; d = 0.30–0.38; *p* < 0.001)
Default	ND		low ripples	45s (↑ 18–34%; d = 0.27–0.51; *p* < 0.001)
			high ripples	440s (↑ 11–14%; d = 0.05–0.21; *p* < 0.001)
Default‐ Dorsal attention	ND		low ripples	50s (↑ 9–16%; d = 0.37–0.67; *p* < 0.01)
Default – Ventral attention	ND		low ripples	50s (↑ 14–19%; d = 0.27–0.41; *p* < 0.01)
Default – Somatomotor	ND		low ripples	50s (↑ 18–28%; d = 0.30–0.45; *p* < 0.001)
			high ripples	80s (↓ 8–17%; d = 0.09–0.21; *p* < 0.001)
Default – Visual	ND		low ripples	430s (↑ 19–31%; d = 0.51–0.78; *p* < 0.01)
Default – Frontoparietal	ND		low ripples	275s (↑ 13–23%; d = 0.27–0.39; *p* < 0.001)
Dorsal attention	ND		low ripples	280s (↑ 22–25%; d = 0.38–0.60; *p* < 0.001)
Dorsal attention – Limbic	ND		low ripples	80s (↑ 31–56%; d = 0.35‐37; *p* < 0.001)
Dorsal attention – Ventral attention	ND		low ripples	345s (↑ 10–23%; d = 0.23‐53; *p* < 0.001)
Dorsal attention – Frontoparietal	ND		low ripples	45s (↑ 14–49%; d = 0.27‐51; *p* < 0.001)
Visual	high ripples	−70s (↓ 20–40%; d = 0.59–0.61; *p* < 0.001)	ND	
Visual – Somatomotor	high ripples	NC (↑ 75–81%; d = 0.92–0.95; *p* < 0.001)	low ripples	225s (↑ 24–39%; d = 0.31–0.51; *p* < 0.01)
Somatomotor	ND		low ripples	50s (↑ 14–21%; d = 0.20–0.47; *p* < 0.001)
Somatomotor‐ Dorsal attention	ND		low ripples	275s (↑ 23–41%; d = 0.44–0.77; *p* < 0.001)
Somatomotor – Frontoparietal	ND		low ripples	50s (↑ 18–35%; d = 0.26–0.49; *p* < 0.001)
			high ripples	485s (↓ 2–14%; d = 0.07–0.13; *p* < 0.01)
Ventral attention – Frontoparietal	ND		low ripples	245s (↑ 4–15%; d = 0.15–0.33; *p* < 0.001)
Frontoparietal	ND		low ripples	380s (↑ 9–27%; d = 0.27–0.39; *p* < 0.001)

**Figure 6:**
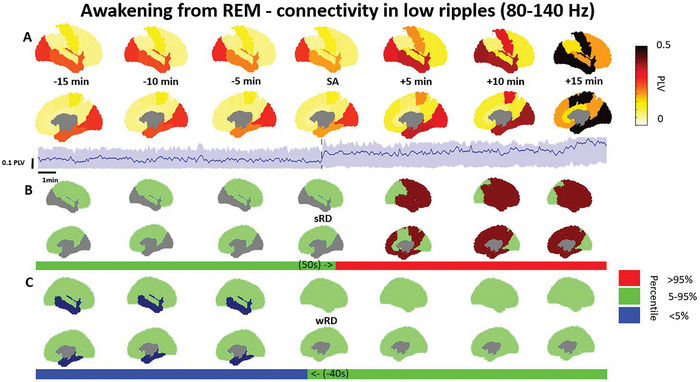
Phase connectivity within networks in the high ripple band increases with awakening from REM. A) Phase locking value (PLV) within each available network in the brain is shown from 15 min before to 15 min after the scalp awakening (SA), along with a network example from every available network. Quartile ranges are represented by the shaded area. The respective intracranial awakening times for sleep reference distribution (sRD) and wakefulness RD (wRD) B) The overall PLV percentile in relation to the sRD within every network complemented by a network example in the bar. C) The overall PLV percentile in relation to the wRD is presented by the bar, with an accompanying network example above. Note: PLV represents the weighted median power within each network. The reference distribution percentiles are based on the weighted median within each network, which are then categorized as >95%, within 5–95%, or < 5%. Continuous PLV examples and comparisons to wakefulness and sleep reference distributions are illustrated for the limbic network. Deviations below the 5% or above the 95% percentiles are deemed significant Networks include: visual, limbic, dorsal attention, ventral attention, somatomotor, and default mode. Data was presented for 12 patients from a total of 513 channels, ranging between 5–43 per network (Full description in Table S1B, Supporting Information).

When awakening from REM sleep, a widespread modest increase in the low ripple band PLV was observed within the limbic network and to a lesser degree, in the default mode, dorsal attention, somatomotor, and frontoparietal networks (**Figure** **6**). This modest change in the relative deviation from sRD ranged between 9–39% (d = 0.20–60; *p* < 0.001) and started late, diverging between 45 to 380 s after the SA. However, only the limbic network differed from the wRD, displaying a relative deviation of 36–40% (d = 0.30–0.38; *p* < 0.001) terminating 40 s prior to the SA, while other networks were not affected. We also observed that between networks, there was a very widespread PLV increase in the low ripple band in most, but not all network pairs. This modest change in the relative deviation from sRD ranged between 2–56% (d = 0.07–78; *p* < 0.01) and started late, diverging between 45 to 430 s after the SA. However, keeping in line with the known similarities between wakefulness and REM sleep, the phase connectivity in most networks did not differ from the wRD during the awakening process. Notable exceptions were the high ripple band, which showed a decrease in the PLV between the limbic, somatomotor, visual, and ventral attention networks during the awakening process. This decrease was mainly apparent in relation to the wRD having a relative deviation ranging between 14–81% (d = 0.21–95; *p* < 0.01). This change terminated between 75 to 455 s prior to the SA, however, some connections never converged back to the wRD. In addition, the PLV within the visual network increased during the process in relation to the wRD, only showing a relative deviation ranging between 20–40% (d = 0.59–61 *p* < 0.01). This change terminated 70 s prior to the SA.

## 3 Discussion

Awakening is a complex heterogeneous process that terminates the sleep period. Here we examined the phenomenon of the awakening process from a local perspective using SEEG from multiple brain regions. Using depth electrodes, we acquired not only high spatial resolution recordings but also high‐quality recordings while avoiding contamination by muscle artifacts as often encountered in scalp EEG recordings during body movements, as typically present during awakenings. We leveraged these inherent advantages of SEEG to examine the awakening process, which for the first time is being evaluated for its high frequency content. This is of particular interest given the tight link between high frequencies and cognitive processes.^[^ ^31^ ^]^ Our results demonstrate: i) the association of fast frequencies in the process of awakening, ii) some regional differences might be influenced by the prior sleep stage, and iii) the spatio‐temporal heterogeneity of awakening from both a spectral and a phase connectivity point of view.

Our results show that high frequencies in the ripple range >80 Hz are associated with the process of awakening (Figures 3, 4, 5, 6). These results are in line with the known association between fast frequencies and cognition,^[^ ^8^ ^]^ as well as consciousness,^[^ ^9^ ^]^ which forms the essence of the transition from sleep to wakefulness. Assessing high frequencies poses challenges without high quality, low noise, and high sampling rate recordings. We suspect that this is the reason that to date, the investigation of awakenings has not been able to capture the contribution of high frequency activity. Nevertheless, when an attempt was made to investigate the sleep‐wake cycle and cognition using SEEG, it revealed the involvement of the low gamma band.^[^ ^32^ ^]^ We observed that both the spectral content (Figures 3 and 5) as well as the phase connectivity of high frequencies (Figures 4 and 6) increased during the awakening process. Our spectral results correspond to a recently described high frequency index which was able to discriminate between NREM, REM, and wakefulness in rats.^[^ ^33^ ^]^ In our case, the spectral content of high frequencies gradually increased during the awakening process. In addition, the connectivity increase during the awakening process aligns with previous findings that showed higher connectivity in wakefulness compared to NREM in the low ripple band using another phase‐based connectivity metric (weighted phase lag index).^[^ ^34^ ^]^ Our results, which demonstrate an association between increased higher frequencies and awakening, align with accumulating evidence suggesting that higher frequencies play a critical role in consciousness.^[^ ^35^, ^36^, ^37^ ^]^ These findings might support the idea that changes in high‐frequency activity are key during the transition from sleep to wakefulness. When NREM parasomnias were investigated using SEEG, it was observed that the interplay between high frequencies (>20 Hz) and delta activity appears to be associated with the level of conscious awareness.^[^ ^38^ ^]^ Furthermore, another recent study that used SEEG to investigate the connectivity of transient high frequencies oscillations in wakefulness and NREM sleep demonstrated a similar widespread synchronization across different regions.^[^ ^39^ ^]^ We opted to use ripple band activity rather than individual ripple events in order to assess the progressive change in the signal in all the frequency bands with a consistent methodology. In addition, it would be difficult to assess just using ripple rates due to their high variability and sparsity of ≈3 ± 3 per min^−1^.^[^ ^39^ ^]^ Our findings of increases in high frequencies might be in line with past investigations into dreaming which also reported such associations.^[^ ^24^ ^]^ suggest shared neural dynamics between awakening and dream‐related processes. In addition, due to the various timing of changes in high frequencies, some of which occur before the scalp awakening when the patient still is behaviorally asleep, and due to the lack of large‐scale cross‐band activity (specifically the absence of changes in the beta band), we believe that our results reflect a real process taking place during the awakening process and are not solely the result of movement artifacts.

Awakenings from NREM and REM sleep display different patterns, with the awakening process being influenced by the prior sleep stage (NREM or REM), both in terms of spectral and connectivity content. Spectrally, awakenings from NREM required changes in multiple bands, with lower bands decreasing and high frequency bands increasing. Awakenings from REM consisted mainly of an increase in the gamma‐ripple bands. Interestingly, awakenings from REM, which are classically hard to distinguish from wakefulness using scalp EEG alone,^[^ ^40^ ^]^ displayed a widespread increase in the ripple bands and only a few changes in lower frequencies (Table 3).

Previous investigations using scalp EEG have shown an increase in delta activity in the first few minutes after awakening from NREM and REM compared to pre‐sleep wakefulness.^[^ ^5^, ^6^ ^]^ In contrast, our results showed a gradual decrease in delta power which converged to wakefulness tens of seconds prior to SA. We suggest that this discrepancy may be explained by differences in definitions of when divergence/convergence are determined. In our study, we employed a stricter definition requiring the signal to be within the 5–95% percentile to be considered as divergence/convergence, as opposed to accepting any statistically significant change. Spatially, we observed changes in the delta band in the frontal, partial, and medial regions. While these results concur with that of previous studies,^[^ ^5^, ^6^ ^]^ we did not have enough sampling of occipital channels to assess the changes in delta previously reported.^[^ ^6^ ^]^ It was also reported that beta activity post‐awakening from REM, and to a lesser extent from NREM,^[^ ^41^ ^]^ was lower compared to pre‐sleep wakefulness. We observed these beta power changes during awakening from NREM in the superior, middle, and orbital frontal gyri, and the anterior part of the inferior frontal gyrus region, as well as during awakenings from REM in temporal parietal regions. In addition, the observation of significant differences in high frequency activity between REM sleep and wakefulness at the moment of awakening aligns with earlier findings from high‐density EEG studies. These studies reported more extensive variations in beta power (12–20 Hz) compared to delta power (1–4 Hz, primarily localized to sensory cortices) during consistent periods of REM sleep versus wakefulness.^[^ ^42^ ^]^ We can speculate that the reason more spectrally wide changes occur in awakenings from NREM might be because NREM is more impaired during NREM^[^ ^43^, ^44^ ^]^ thus requiring more changes to return to full consciousness. The observed increase in high frequency activity during awakening may be linked to cognitive recovery and sleep inertia. Disruptions in this activity could underlie the cognitive impairments of sleep inertia. The availability of multiple nights with a clear morning awakening of the same patient using sEEG was not available. We are unable to compare directly NREM and REM due to the lack of availably of awakenings from both stages within the same patient. Thus, future research is needed to assess the full effect of the differences between awakening from NREM and REM.

Recently, more evidence has accumulated to support the role of connectivity within the different brain networks in facilitating transitions between sleep and wakefulness.^[^ ^45^, ^46^ ^]^ We wanted to explore these differences during the awakening process. From a phase connectivity perspective, we found that awakening from NREM showed a focal change almost exclusively in the low and high ripple bands (Table 2). This was most apparent within network phase connectivity in the somatomotor, default mode network, and dorsal attention networks (Figure 4), as well as in the connectivity between these networks. However, not all networks participated, with some networks never displaying any significant changes during the process. When awakening from REM sleep, phase connectivity changes were mainly observed in the low ripple band (Table 4) and were prominent mainly in the limbic network (Figure 6), as well as other networks such as the default mode network. We opted to show the most prominent results for each analysis. In Figure 6, we present the results from the low ripple band. Our phase connectivity results align with EEG‐fMRI findings, showing that awakenings result in a different correlation connectivity pattern depending on the prior sleep stage,^[^ ^15^, ^17^, ^47^ ^]^ particularly within the default mode network.^[^ ^15^, ^48^ ^]^ In addition, we expanded these findings by exploring higher frequencies that are not accessible using fMRI and were able to demonstrate their role in the waking process. We observed a similar upward trend in both power and phase‐based connectivity in the ripple band; however, this similarity did not extend to other bands. Further research is needed to elucidate this connection, which has also been observed for transient high frequency oscillations.^[^ ^39^ ^]^ On top of that, we focused on morning awakenings rather than awakenings from a short nap as they are more representative of the full phenomena of human sleep. Two recent seminal studies^[^ ^13^, ^14^ ^]^ on awakening using EEG‐fMRI discovered that the thalamocortical connection preceded the scalp awakening. However, we were unable to confirm these findings due to a lack of thalamic recordings in our cohort. The more spectrally restricted signature of REM awakening, focused on increases in high frequencies, may suggest that different states of consciousness require distinct alterations to transition back to regular wakefulness. Future research is needed to further clarify the potential effects of circadian rhythm on spectral and connectivity dynamics, particularly given its demonstrated influence on sleep inertia.^[^ ^49^, ^50^, ^51^ ^]^

Awakening is a heterogeneous process. We observed a high degree of spatial and temporal variability during awakenings (Tables 1, 2, 3, 4). This spatial heterogeneity adds information on the regional variability of wakefulness^[^ ^52^ ^]^ and sleep.^[^ ^11^ ^]^ However, the temporal heterogeneity that we observed demonstrates that the global phenomenon of the transition between sleep to wakefulness, as reflected using scalp EEG, is not uniform, and does not follow a clear gradient as seen, for example, for NREM to REM sleep transitions.^[^ ^53^ ^]^

The boundaries between sleep and wakefulness appeared fluid. When evaluating the awakening process, there are two ways to examine it: one as a temporal change from sleep, i.e., comparing it to the immediate time prior to wakening, and second, as its difference to full wakefulness, i.e., comparing it to resting state from wakefulness. In order to address both of these questions, we performed a continuous comparison at each time point to both the sRD from −15 to −10 min prior to the SA, and to the wRD taken from the previous day. This allowed us to assess both the signal transition from sleep to wakefulness, as well as to determine when we returned to the bounds of normal wakefulness. Throughout the results, we noticed a discrepancy between the IAs of the wRD and the sRD. This may be explained by an expected overlap in the boundaries between all ranges of wakefulness activity and the boundaries of sleep. We postulate that these edges might reflect the overlapping boundaries of the different states of consciousness. This might be related to the notion of local regulation of consciousness, in which different states such as sleep and wakefulness can co‐occur in different brain regions at the same time.^[^ ^12^, ^54^, ^55^ ^]^ The later can also explain our observation that some brain regions never diverged from wakefulness or never converged back to it. We suggest that higher frequencies, although not exclusively, might play a role in facilitating the transition between sleep and wakefulness without ever exceeding any boundaries. In addition, awakening from REM did not converge within the 30 min to the wRD in one area (medial and basal temporal region), perhaps as more time might be needed for it to return back to normal wakefulness, potentially contributing to the behavioral phenomena of sleep inertia. In addition, wakefulness^[^ ^56^ ^]^ and arousal states^[^ ^57^ ^]^ are heterogeneous processes with many different patterns of activity occurring within them, as reflected in the EEG. This variability may cause the wRD to have a wide range of possible activities since the segments used were from daily life rather than a controlled environment. This, in turn, suggests that it is more difficult to diverge from our wRD, especially with a strict 5–95% threshold. This may explain why some regions showed changes in relation to the sRD but did not show changes in relation to the wRD. Future research is needed to better control for different sensory inputs, times, and environments of the wakefulness distribution in order to further parse which activities diverge and which do not. Variability between patients was visible in different regions and networks at specific time points. Future research with a larger sample size is needed to untangle these differences with respect to visual and auditory inputs.

Our study has the following limitations. One inherent issue of SEEG is its limited sampling of the brain, which means that we do not have complete brain coverage for each sleep stage leading up to awakenings. To address this limitation, we aggregated the results across all available channels obtained from all patients and then grouped them using an atlas. However, this still limits our ability to directly compare NREM and REM awakenings as we have different coverage with their respective patients, which may not share all regions and networks. We were also unable to control directly for the circadian rhythm when selecting for the control segment however we chose them from the same part of the day. Moreover, this approach is limited to subcortical‐cortical connections such as thalamo‐cortical, which are not usually implanted. In addition, this aggregation may lead to under‐detection of small effects, as they are more likely to be overshadowed by inter‐patient variability. Obtaining human SEEG recordings is restricted to epileptic patients undergoing SEEG during pre‐surgical evaluations in a clinical setting. Also, patients are usually on antiseizure medication that can impact sleep architecture. Finally, epilepsy itself causes changes to the EEG beyond epileptic spikes and can go along with comorbid sleep disorders. To address this concern, we carefully deselected all channels with abnormal EEG activity following a strict protocol to address this issue as effectively as possible. To the best of our knowledge, all of the awakenings are spontaneous awakenings. That being said, data were recorded in a clinical epilepsy monitoring unit setting and not a sleep laboratory so that external noise resulting in some of the awakenings cannot be totally excluded. However, we believe that finding reproducible effects speaks against the fact that different types of awakenings were included. Direct comparisons between NREM and REM within individual patients were not feasible due to the study's limited duration, which allowed for only a single night of sleep analysis. Comparisons across patients were also constrained by the inherent sampling limitations of SEEG, as each implantation is individually planned to meet the specific clinical needs of each patient. This variability in electrode coverage prevents consistent cross‐patient comparisons. As a result, the absence of findings should not be interpreted as negative results but rather a call for future studies to gather more data on the awakening process in these missing regions. Finally, future work might be needed with multiple nights of recordings in the same patients, with both awakenings from NREM and REM, to directly compare them within the same patients and region. Lastly, an avenue that we could not explore here is the influence of dream content on the awakening process; both REM and NREM contain dreams even if their frequency of recall varies,^[^ ^26^ ^]^ yet their dream content differs.^[^ ^58^ ^]^ The primary limitation of SEEG lies in its restricted coverage, as it allows access to only a limited portion of the brain in each patient. However, recent pioneering work has demonstrated the feasibility of combining SEEG with high‐density EE,^[^ ^59^, ^60^, ^61^, ^62^, ^63^ ^]^ achieving comprehensive brain coverage. Future research stands to gain significantly from adopting this innovative yet challenging methodology to investigate neural dynamics during awakening. By leveraging this combined approach, researchers can better assess long‐range changes in spectral power and connectivity.

In summary, the process of awakening is complex and can differ in both timing and location. Nonetheless, high frequency bands appear to be associated with the process of awakening from both NREM and REM sleep across multiple brain regions. Our findings indicate that, while scalp recordings can provide a global impression of the awakening, local recordings reveal interactions between regions that can occur several minutes before or after the scalp awakening. Finally, we demonstrated that the prior sleep stage can influence the local spectral and connectivity activity patterns during the awakening.

## 4 Experimental Section

### Patient Selection

Eighteen consecutive patients with drug‐resistant focal epilepsy (7 female; age = 36.9 ± 11.5 years, Table. S6, Supporting Information) who underwent combined SEEG and PSG recordings as part of their presurgical investigation at the Montreal Neurologic Institute and Hospital between January 2013 and June 2022 fulfilled selection criteria (see flowchart of patient selection in chart. S1, Supporting Information). Inclusion criteria were clear awakenings without falling back to sleep, age >15 years, recordings composed of at least 5 healthy non‐epileptic intracranial channels outside the seizure‐onset‐zone (SOZ), recordings with a minimum duration of 10 min after the morning awakening, availability of a post‐implantation imaging for accurate anatomical localization of the individual channels, and absence of seizures in the sleep cycle prior to the morning awakening. All awakenings corresponded to spontaneous awakenings. Forced awakenings were not studied, as the interest was in the natural process of awakening. Patients without a clear final morning awakening, defined as no N1 in the 15 min after awakening, were excluded, as well as patients who did not have stable sleep, defined as ≥3 N1 epochs in the 5 min prior to awakening. The study was approved by the Montreal Neurological Institute and Hospital Review Ethics Board (2014‐183).

### Intracranial EEG and Scalp EEG Recordings

An average of 11.5 ± 4.3 depth MNI SEEG electrodes (9 contacts of 0.5–1 mm, distance between contacts 5 mm; 4 patients) or DIXI SEEG electrodes (10–15 contacts of 2 mm, distance between contacts 1.5 mm; 14 patients) were stereotactically implanted in every patient. Scalp EEG was obtained simultaneously with subdermal thin wire electrodes at positions Fz, Cz, Pz, F3, C3, P3, F4, C4, and P4, and additional electrodes for electrooculogram and chin electromyogram were applied during the night of the sleep recording. The sleep recording was chosen at least 72 h after implantation to avoid the effects of anesthesia as frequently seen in the first days after implantation.^[^ ^64^ ^]^

EEGs were recorded using the Harmonie EEG system (Stellate) from 2013 to 2017 and the Neuroworkbench EEG system (Nihon Kohden) after 2017. Recordings were acquired with a common reference (epidural electrode fixed in the bone, far from the epileptic field). Hardware filter settings were 0.1 Hz for the high pass filter and 500 (Stellate) or 600 Hz (Nihon Kohden) for the low pass filter. All recordings were sampled at 2 kHz.

### Channel Selection and Classification

Each SEEG contact was clinically assessed by an epileptologist (B.F.), and only normal healthy channels were selected. Healthy channels were defined as channels situated within healthy tissue, as determined by MRI scans, positioned away from the area where seizures originate, and consistently free from interictal epileptic discharges and significant slow‐wave anomalies throughout the circadian cycle. This assessment was based on the comprehensive implantation report and a detailed examination of one night's sleep by a board‐certified electrophysiologist. Channels in the white matter were further excluded,^[^ ^52^ ^]^ resulting in 695 channels (38.6 ± 33.4 per patient) for analysis (Figure 1).

Registration of the electrodes was done in a similar manner to our previous work^[^ ^52^ ^]^ where Minctools and the IBIS framework^[^ ^65^ ^]^ were used. This involved first linearly aligning the CT/MRI images taken after electrode implantation, which display the locations of the electrodes, with the pre‐implantation MRI scans (preMRI) for each patient. Following this, the preMRI images were non‐linearly aligned with the ICBM152 non‐linear symmetric brain model.^[^ ^66^ ^]^ Through this combined transformation process, it was possible to accurately estimate the electrode positions within a unified stereotaxic space. Channels were then classified into anatomical regions using the MICCAI atlas^[^ ^67^ ^]^ reduced to 17 regions^[^ ^68^ ^]^ combining hemispheres that offer sufficient anatomical coverage and granularity, and into brain networks using the Yeo 7 networks atlas.^[^ ^69^ ^]^ The Yeo 7 network atlas has recently been applied in sleep research investigating sleep dynamics using fMRI,^[^ ^70^, ^71^ ^]^ and offers a functional way to assess connectivity results. Registration of the electrode positions was done independently for the MICCAI atlas and the Yeo 7 networks using a 5 mm radius growing region. Finally, patients were grouped using the last sleep stage prior to their awakening, either from REM or NREM sleep. Only regions from ≥3 patients and ≥5 channels and networks that had ≥3 patients and ≥5 channels and at least two different structures were analyzed for spectral and connectivity content (Figure 1; Figures S1, S2, and Table S1, Supporting Information).

### Sleep Scoring and Segment Selection

Sleep scoring was done by a board‐certified neurophysiologist (B.F.) according to the American Academy of Sleep Medicine criteria.^[^ ^72^ ^]^ The exact time of the SA was marked independent of the SEEG. Final morning awakenings (6:30–9:00 AM) were selected for each patient based on the SA time. The awakening time was determined as “final” if the following wake period did not contain any N1 epochs after the SA. This time point was then used as the reference awakening throughout the paper when examining changes in SEEG channels. 15 min before and after the SA time were extracted in order to assess the full extent of the awakening as a complete process. 15 min were chosen as in the clinical setting post‐awakening the patients were disconnected shortly after.

Baseline segments were selected as references to compare each time point during the awakening process. Two reference distributions were used: i) using 10 min wakefulness segments taken from the evening before the sleep recording (>2 h before sleep onset), and ii) segments from the sleep period between 10 to 15 min prior to the SA. Segments were visually inspected and selected by a board‐certified neurophysiologist (C.A.). Channels without epileptic spikes and segments that were >90 min away from seizures were selected. Channels without spikes were defined as non‐epileptic by a board‐certified epileptologist. In addition, spikes were quantitatively detected, and selected a cut‐off of 3 spikes per 10 min, which corresponds to the false positive rate of the automatic detector.^[^ ^73^ ^]^ This sleep segment overlapped intentionally with the ‐15 min to +15 min from the SA in order to evaluate the relative evolution of the awakening itself. Finally, to avoid any spurious spiking activity, all channels were checked automatically for epileptic spikes detection,^[^ ^74^ ^]^ and corresponding detected signals were discarded using a window of ‐100 to 100 ms around the peak of each spike for both the awakening and control segments. These selections accounted for less than 1% of the total signal. To definitively rule out any effects of removing these signal periods, an identical analysis was also performed not rejecting the quantitatively identified spurious spiking activity.

### Preprocessing and Analysis

The SEEG signals were analyzed using a common average montage, which incorporated all non‐SOZ and visually detected artifact‐free channels for both power and connectivity analysis. Signals were filtered with a Butterworth band‐pass filter ranging from 0.3 to 300 Hz. The awakening process was analyzed starting 15 min before the awakening, as detected on the scalp EEG, and continuing until 15 min after. The analysis was done over 5 s segments, without overlap, throughout the 30 min signal. 5 s windows were chosen as a window in order to guarantee reliable spectral^[^ ^75^ ^]^ and connectivity^[^ ^76^ ^]^ estimates in both high as well as low frequencies. RD were also constructed to extract normative distributions when characterizing the awakening process, using wakefulness control segments from between 4–8 PM of the evening before the sleep recording and a sleep segment from −15–10 min before the awakening (Figure 2A i). To enhance the reliability of these baseline segments, the segments were bootstrapped using a block design. To do so, it was sampled 10 000 times with the replacement of raw signals in 5 s blocks from the 10 min of wakefulness or the 5 min of sleep (Figure 2A i). To assess the potential impact of circadian rhythms on the wakefulness baseline, an additional baseline curated by an independent epileptologist (G.R.) not involved in the original data selection was included. This baseline, selected from quiet wakefulness periods between 8 AM and 12 PM on the day of the sleep SEEG recording, was used to compare IA times in relation to wakefulness. The spectral and connectivity properties of the awakening process were investigated during the selected 30 min period of the awakening process (Figure 2A ii). The analysis was conducted for the following high frequency bands: low gamma (30–50 Hz), high gamma (50–80 Hz), low ripple (80–140 Hz), and high ripple (140–200 Hz). This distribution was chosen to ensure equal representation in the gamma and ripple bands. Furthermore, traditional frequency bands were also analyzed for comparison purposes: delta (0.5–4 Hz), theta (4–8 Hz), alpha (8–13 Hz), and beta (13–30 Hz). Artifacts were visually rejected as well as rejected using automatic amplitude base artifacts (>5 standard deviations). The spectral analysis was executed using a Morlet wavelet transform on each 5 s segment without overlap. A Morlet transform featuring a 1 Hz central frequency and a duration of 3 s was opted to strike a balanced trade‐off between temporal and frequency resolution.^[^ ^77^ ^]^ The outputs from the Morlet wavelet transform were treated separately for each band. This spectral analysis was carried out for every available normal channel for each patient. For visualization, only the power was normalized between 0 and 1 across the brain, facilitating improved visualization of both temporal and regional differences. Connectivity analysis followed, where PLV was estimated for each pair of available normal channels within each patient.^[^ ^78^ ^]^ This was done for each pair within each available network and for each pair that was paced in two different networks. This was done to assess the connection strength within every network as well as the strength of the connection between different networks. The PLV was used in order to capture the phase connectivity which was insensitive to the amplitude due to the variability of amplitudes that can occur in SEEG.^[^ ^79^ ^]^ The PLV was computed in each frequency band separately as $PLV_{i,j,f}=\frac{1}{T}\;\left|\;\sum_{t=1}^{T}e^{-1\left(\phi_{i}(t\right)-\left(\emptyset_{j}(t\right))}\right|$ where *i*, *j* are channels, f is the frequency band of interest, T is the signal length and ϕ is the instantaneous phase estimated as the angle of the analytical signal from the Hilbert transforms of both signals. Subsequently, the power or PLV signals estimated every time point of interest during the awakening process were compared to both the sRD and the wRD in terms of percentiles relative to that RD (Figure 2A iii). This process generated three 30 min time series: 1) the original power or PLV signals, 2) the signal represented as a percentile when compared to the sRD, and 3) the signal represented as a percentile compared to the wRD. These time series were then pooled for all patients, for each channel or channel pair (Figure 2A iv). These channels or channel pairs were grouped using a weighted median over all the channels in the region/network. The weights, which add up to one, are defined for each power/PLV as: $w=\frac{1}{NR_{s}}$ where *w* = weight of the specific channel (or channel pair for PLV), *N* = number of patients contributing to a region/network, and *R_s_* = number of channels contributing to the region/network for a specific patient. The power/PLV values and the weights were sorted separately in an ascending order and then a cumulative sum of the weights was calculated until it reached 0.5. The median weighed power/PLV was defined as the matching power/PLV value of the corresponding index at 0.5. This approach was adopted to account for the relative contribution of each patient. Finally, these time series were compared throughout the awakening process, considering both the original power and PLV as well as a categorized normalized time series in relation to the sRD and wRD (Figure 2A v). The latter was achieved by categorizing the percentile time series into three bins with respect to the corresponding RD: 1) significantly larger, percentile >95% (red), 2) within 5–95% (green), and 3) significantly smaller, percentile < 5% (blue). A continuous description of the awakening process from different perspectives is provided (Figure 2B). First, the original power/PLV values were presented every 5 min for all available regions or networks, along with an example of one region/network (Figure 2B i). Second, a description was presented every 5 min of the categorized percentiles compared to the sRD (Figure 2B ii) and the wRD (Figure 2B iii) for all available regions or networks, as well as a continuous example from a selected region/network. Two times for IA were then estimated; the first one being the divergence from sleep (when compared to sRD) and the second one being the convergence to wakefulness (when compared to wRD). These IA times reflect the moment the signal either diverges from the sRD or converges to the wRD. The IA times were identified as the initial time instance after more than three continuous epochs (90 s) during which the percentile was either falling outside (for sRD) or inside (for wRD) the 5–95% boundaries of the corresponding RD. Strict 5–95 percentile thresholds were chosen to ensure that only stable non‐spurious changes were considered. Therefore, the IA times represent the first stable instance of convergence to wakefulness or divergence from sleep. Note that the binarized data was only used for the purpose of determining the IA time. The subsequent analysis for the statistical assessment of the differences, effect sizes, and magnitude of change was conducted using the original power and PLV. All the analysis was done using MATLAB 2023a, and signal processing was done using the Brainstorm toolbox.^[^ ^80^ ^]^

### Statistical Analysis

Six patients awakening from NREM sleep and twelve patients awakening from REM sleep were analyzed. Each time point in the awakening process was evaluated in relation to the sRD and wRD, by performing a continuous statistical comparison (Figure 2C). 90 s segments were analyzed every 5 min before the wakefulness IA and after the sleep IA. This analysis was conducted on the channel level for all the available channels in the region or network. The median power/PLV of the awakening signal was compared to the median RD using a two‐side paired Wilcoxon signed‐rank test, with a false discovery rate (FDR) correction. The effect size was estimated using paired Cliff's Delta. No further transformations to the data were applied, and no datapoints were removed. This was done once for all the time points prior to the wakefulness IA and then for all the time points after the sleep IA. Data was presented as median and range as distributions were not always normal. If the difference achieved significance at *p* < 0.05 The magnitude of the change was then assessed by defining the “relative deviation”. This was calculated as the weighted median of the percentage difference on all channels between the awakening signal and the median RD. In other words, for each channel, the extent to which it differed from the RD was assessed and then aggregated it using a weighted median. Comprehensive tables detailing the IA times for the wakefulness IA and the IA were provided for all significant results, i.e., when a band or an area was not present it means no significant results were observed (full results are provided in Tables S2–S5, Supporting Information). For each one, the ranges of Cliff's Delta and the corresponding relative deviations from both the sRD and the wRD for the spectral and connectivity analyses were provided for each available region or network. Note that NREM and REM sleep were not directly compared as only one type of awakening was selected per patient. The awakening process observed on the scalp was also compared to each available region. For this, a single scalp channel (either Cz or Fz) was selected based on visual assessment to ensure minimal muscle‐related artifacts. This channel's data was then compared to the median power of each region at each time point using Pearson correlation. The median coefficient and the FDR corrected p values were reported (Tables S7 and S8).

### Ethical Statement

The study was approved by the Montreal Neurological Institute and Hospital Review Ethics Board (2014‐183). As this research was retrospective, informed consent was waived by the MNI Director of Professional Services.

## Conflict of Interest

The authors declare no conflict of interest.

## Author Contributions

T.A., C.G., and B.F. conceptualized the project and experimental design. T.A. performed the analysis. G.R, C.A, F.D, and B.F. contributed to the curation of data. T.A, B.F., and C.G. participated in the discussion of the methodology, mathematical formulation, and results of the study.

## Supporting information



Supporting Information

## Data Availability

The data that support the findings of this study are available on request from the corresponding author. The data are not publicly available due to privacy or ethical restrictions.
