# Supplementary material for: The Awakening Brain is Characterized by a Widespread and Spatiotemporally Heterogeneous Increase in High Frequencies
Source: Adv Sci (Weinh). 2025 Mar 24;12(19):2409608. doi: 10.1002/advs.202409608 (PMC12097024; doi:10.1002/advs.202409608)
Supplement: Supplementary file 1 — Supporting Information [file ADVS-12-2409608-s001.docx]

**Supplementary**

**Chart S1**


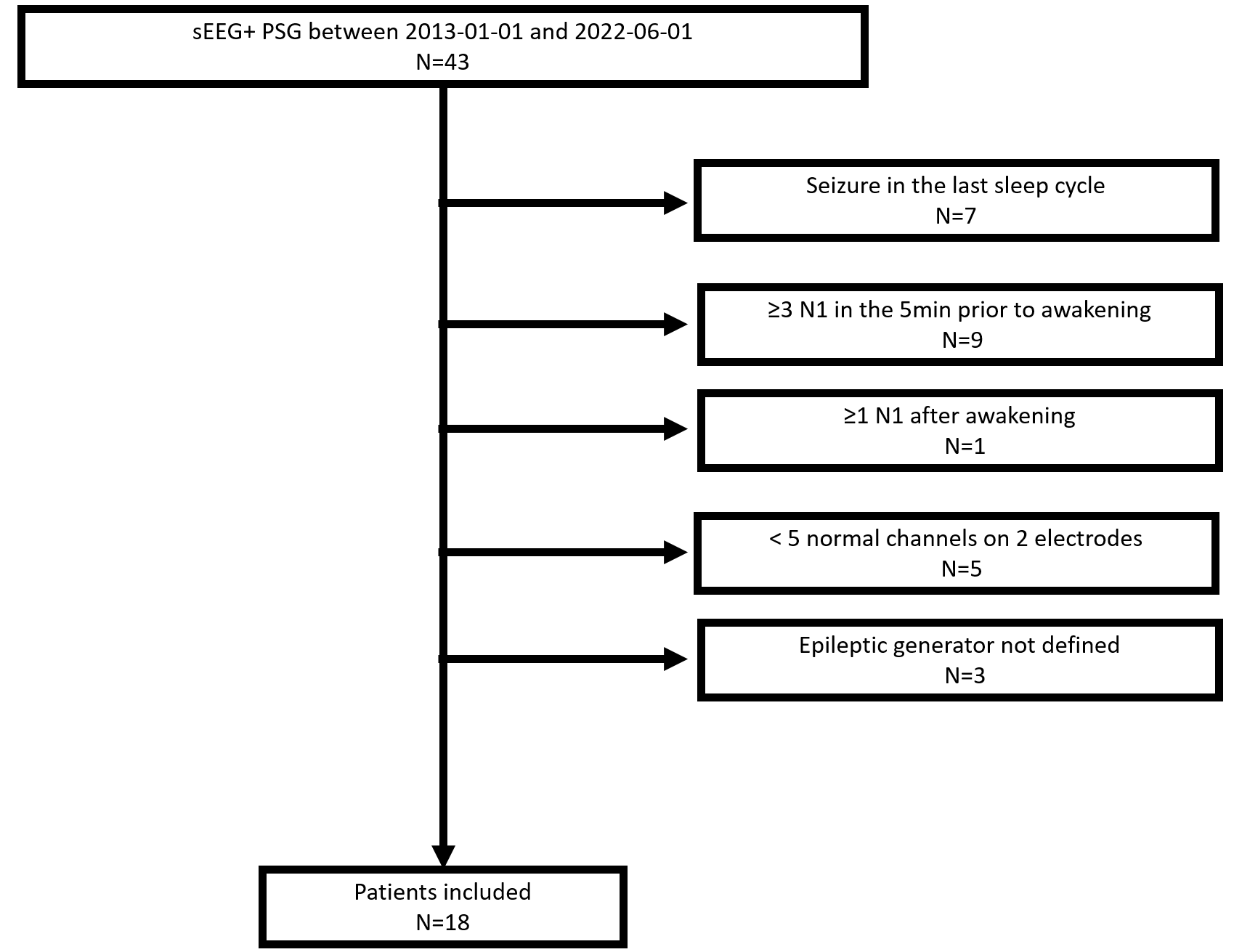


Chart S1 Flowchart of the patient selection process.

**Figure S1**


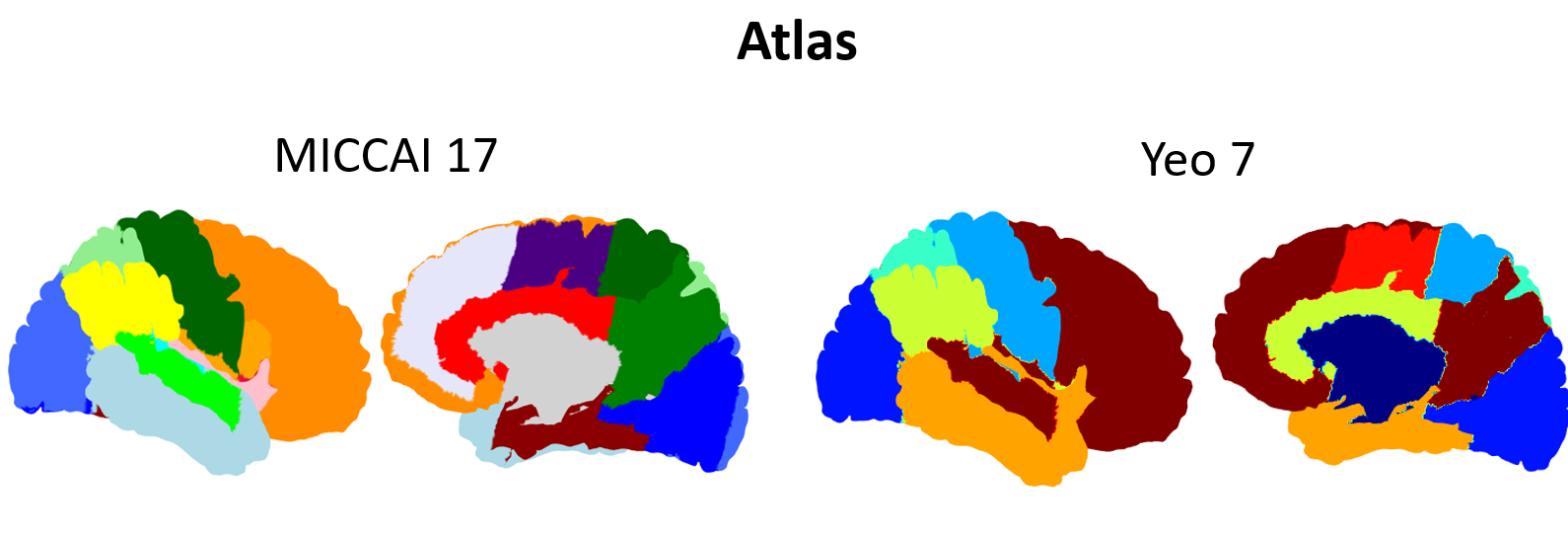


**Figure S1.** MICCAI17 and YEO atlases illustration. * MICCAI regions (medial occipital lobe: blue, lateral occipital lobe: royal blue, medial and basal temporal region: light blue, transverse temporal gyrus and planum temporale: cyan, pre- and postcentral gyri: dark green, medial parietal lobe: green, superior parietal lobule: light green, superior temporal gyrus: lime, supplementary motor cortex: indigo, medial frontal cortex (including medial segment of superior frontal gyrus): lavender, inferior parietal lobule: yellow, central operculum and opercular part of inferior frontal gyrus: orange, superior, middle and orbital frontal gyri and anterior part of inferior frontal gyrus: dark orange, insula: pink, middle and inferior temporal gyrus: dark red, temporal pole, and planum polare, anterior and middle cingulate gyrus: middle light green, frontal operculum: light blue, medial and basal temporal region: red, transverse temporal gyrus and planum temporale: crimson) **Yeo 7 networks (visual: blue, somatomotor: light blue, dorsal attention: light green, ventral attention: lime, limbic: orange, frontoparietal: red, default mode: red).

**Figure S2**


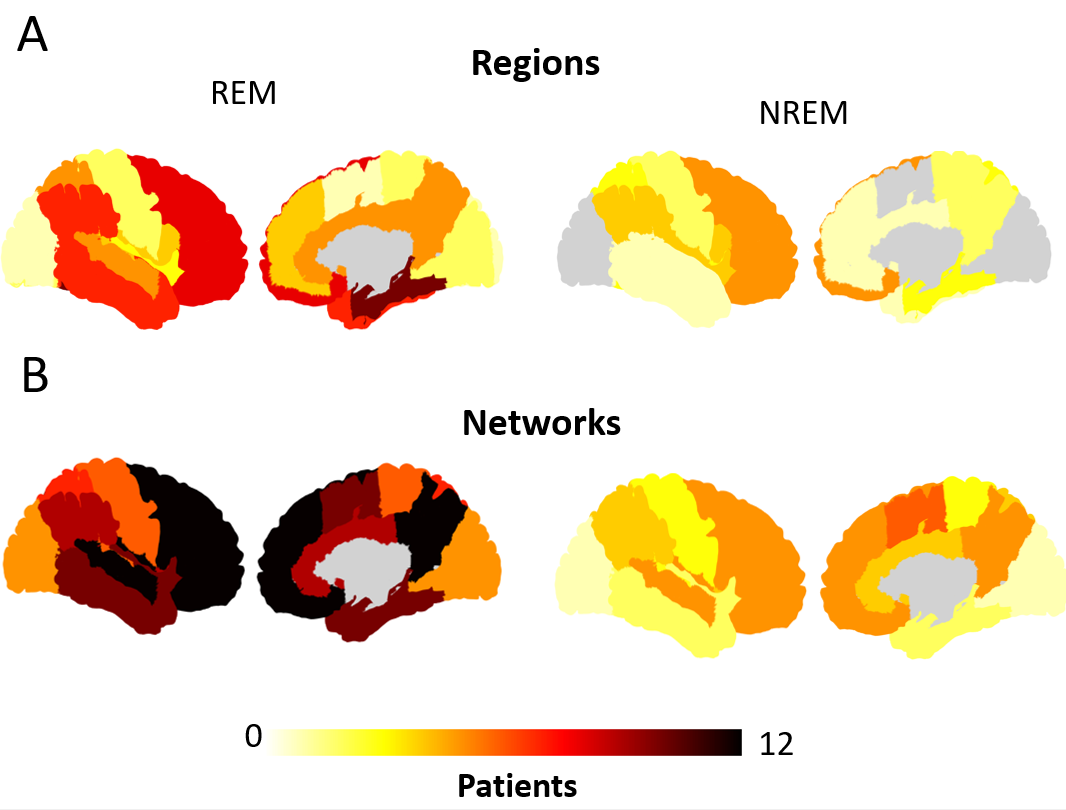


**Figure S2.** Available coverage in the regional and network atlases. (A) Regional coverage in the MICCAI 17 anatomical regions atlas by the number of patients available in each region (B) Regional coverage in networks in the Yeo 7 atlas by the number of patients available in each network.

**Figure S3**


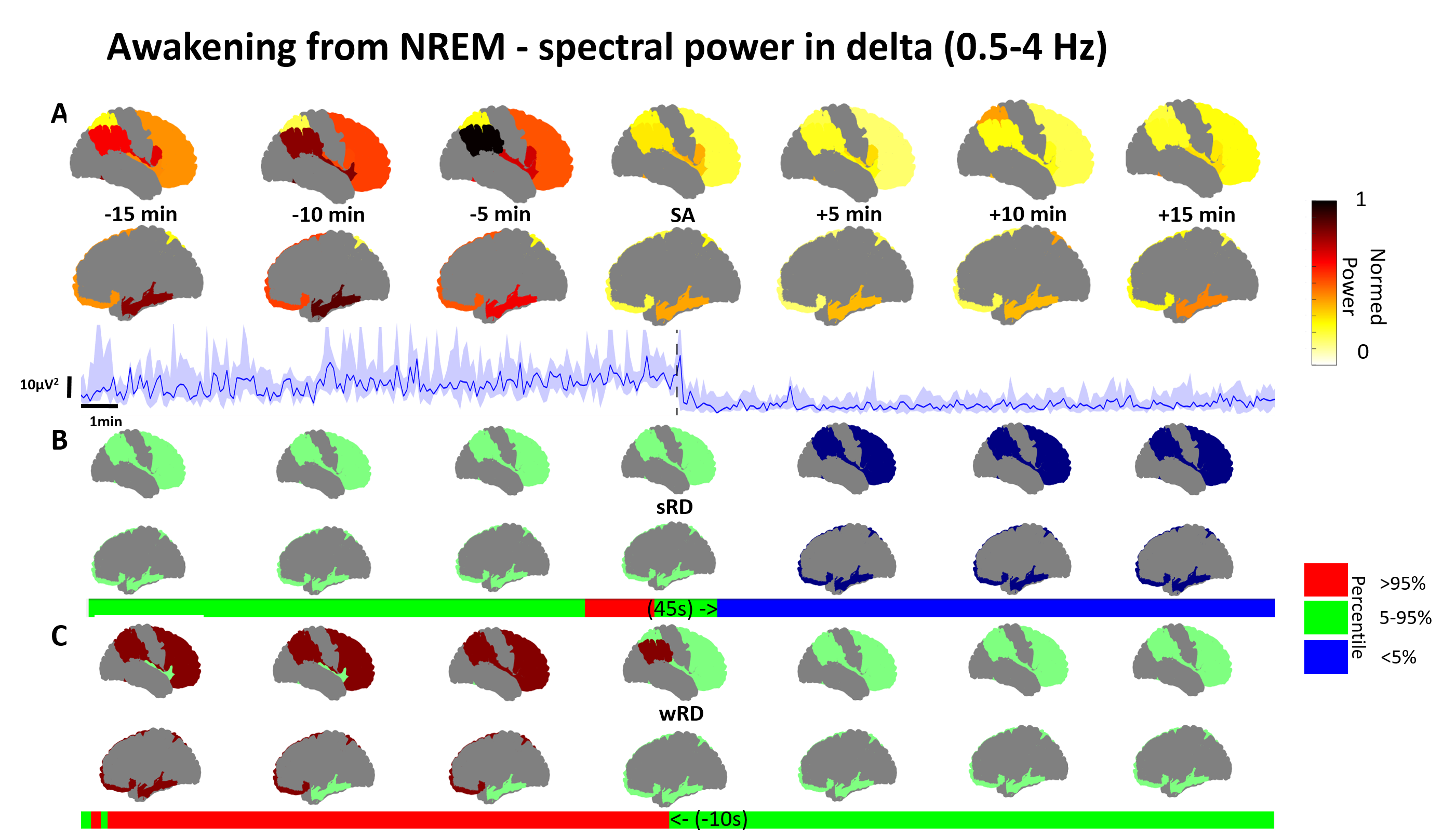


**Figure S3.** Spectral power in the delta band increases upon awakening from NREM sleep. (A) Power from every available region across the brain is shown from 15 min before to 15 min after the scalp awakening (SA), along with a regional example of the power with a quartile range as the shaded area, and the respected intracranial awakening times for sleep reference distribution (sRD) and wakefulness RD (wRD) (B) The power's percentile, in relation to the sRD, is presented for every brain region, complemented by the same regional example. (C) The power's percentile, in relation to the wRD, is detailed for each brain region, with the same regional example as a bar. Note: Power represents the weighted median power in each region, normalized across the entire brain. The reference distribution percentiles are based on the weighted median in each region, which are then categorized as over 95%, within 5-95%, or below 5%. Continuous power examples and comparisons to wakefulness and sleep reference distributions are illustrated for the superior, middle, and inferior frontal regions. Deviations below the 5% or above the 95% percentiles are deemed significant. Included regions are: superior, middle, and orbital frontal gyri and anterior part of inferior frontal gyrus, the insula, the central operculum, opercular part of inferior frontal gyrus, the superior parietal lobule, the middle and inferior temporal gyrus, temporal pole, planum polare, and the Inferior parietal lobule. Data presented for 6 patients from a total of 118 channels, ranging between 15-32 per region (Full description in Table S1 A).

**Table S1**

**A**

| **MICCAI 17 Regions** | **REM** | **NREM** |
| --- | --- | --- |
| Medial occipital lobe | 14 (2) |  |
| Lateral occipital lobe | 4 (1) |  |
| Medial and basal temporal region | 32 (7) | 2 (1) |
| Medial parietal lobe | 20 (5) | 10 (2) |
| Superior parietal lobule | 22 (5) | 17 (3) |
| Superior temporal gyrus | 24 (5) | 9 (1) |
| Supplementary motor cortex |  | 1 (1) |
| Medial frontal cortex (including medial segment of superior frontal gyrus) | 16 (4) | 3 (1) |
| Inferior parietal lobule | 58 (7) | 19 (4) |
| Central operculum and opercular part of inferior frontal gyrus | 21 (3) | 15 (4) |
| Superior, middle, and orbital frontal gyri and anterior part of inferior frontal gyrus | 118 (7) | 32 (6) |
| Insula | 18 (3) | 17 (4) |
| Middle and inferior temporal gyrus, temporal pole, and planum polare | 127 (9) | 18 (3) |
| Anterior and middle cingulate gyrus | 20 (4) | 12(2) |
| Frontal operculum | 9 (4) | 8 (2) |
| Medial and basal temporal region | 32 (7) |  |
| Transverse temporal gyrus and planum temporale | 8 (4) | 5 (1) |
| Pre- and postcentral gyri | 11 (2) | 14 (2) |

**B**

| **Yeo Network** | **REM** | **NREM** |
| --- | --- | --- |
| Visual | 32 (5) {3} | 1 (1) {1} |
| Somatomotor | 47 (6) {6} | 43 (3) {7} |
| Dorsal Attention | 61 (7) {7} | 28 (4) {5} |
| Ventral Attention | 53 (9) {8} | 43 (5) {10} |
| Limbic | 58 (10) {5} | 5 (2) {3} |
| Frontoparietal | 113 (9) {8} | 42 (7) {6} |
| Default mode | 149 (11) {9} | 17 (5) {7} |

**Table S1.** Amount of available channel and patients in every (A) MICCA 17 region (B) Yeo7 networks *labeled as # channels (#patients) {#regions MICCAI17}

**Table S2**

| Region | | NREM | | | | |
| --- | --- | --- | --- | --- | --- | --- |
|  |  | Relative deviation from wRD | | | Relative deviation sRD | |
| Inferior parietal lobule | delta | | -30s (↑ 77-81%; d=.77-86; p<.001) | delta | | 45s (↓95-270%; d=.59-.80;p<.001) |
|  | theta | | -40s (↑ 55-61%; d=.47-.49; p<.001) | theta | | 350s (↓ 77-83Y%; d=.23-.33; p<.01) |
|  | alpha | | (d=.11; p=.7) | alpha | | (d=.03-.11;p=.14-.56) |
|  | beta | | (d=.14; p=.09) | beta | | (d=.04; p=.44) |
|  | low gamma | | (d=.2; p=.09) | low gamma | | 50s (↑ 24-33%; d=.19-.31;p<.01) |
|  | high gamma | | (d=.13; p=.25) | high gamma | | (d=.1; p=.13) |
|  | low ripple | | (d=.22; p=.04) | low ripple | | 200s (↑ 35-47%; d=.25-.35; p<.01) |
|  | high ripple | | (d=.17; p=.25) | high ripple | | 150s (↑ 23-44%; d=.26-.66; p<.001) |
| Central operculum and opercular part of inferior frontal gyrus | delta | | -65s (↓ 30-63%; d=.35-.45; p<.001) | delta | | 215s (↑ 124-165%; d=.46-.53; p<.01) |
|  | theta | | (d=.16; p=.35) | theta | | (d=.14; p=.35) |
|  | alpha | | ND (d=.02-.09;p=.22-1) | alpha | | ND (d=.05-.15;p=.33-.68) |
|  | beta | | -45s (↓ 39-59%; d=.16-24; p<.001) | beta | | (d=;p=) |
|  | low gamma | | (d=.09; p=.27) | low gamma | | (d=.04; p=.84) |
|  | high gamma | | (d=.08; p=.5) | high gamma | | (d=.19; p=.47) |
|  | low ripple | | (d=.15; p=.02) | low ripple | | 230s (↑ 13-22%; d=.03-.26; p<.001) |
|  | high ripple | | -40s (↓ 55-60%; d=.05-.08; p<0.01) | high ripple | | 235s (↑ 14-34%; d=.12-.16; p<.01) |
| Superior, middle, and orbital frontal gyri and anterior part of inferior frontal gyrus | delta | | -30s (↓ 40-46%; d=.39-.48; p<.001) | delta | | 45s (↓ 118-165%; d=.38-.55; p<.001) |
|  | Theta | | (d=.13; p=.77) | theta | | (d=.07; p=.52) |
|  | alpha | | ND (d=.01-.16;p=.44-1) | alpha | | ND (d=.01-.25;p=.23-.85) |
|  | beta | | -45s (↓ 39-59%; d=.16-24; p<.001) | beta | | 30s (↑ 11-25%; d=.03-.13; p<.01) |
|  | low gamma | | (d=.08; p=.13) | low gamma | | 65s (↑ 9-13%; d=.04-.09;p<.01) |
|  | high gamma | | (d=.08; p=.10) | high gamma | | 230s (↑ 9-19%; d=.08-.09; p<.001) |
|  | low ripple | | (d=.17; p=.05) | low ripple | | 190s (↑ 15-39%; d=.14-.23; p<.01) |
|  | high ripple | | -40s (↓ 62-69%; d=.38-.52; p<.001) | high ripple | | 50s (↑ 17-30%; d=.38-.49; p<.001) |
| Insula | delta | | ND (d=.02-.24;p=.1-.45) | delta | | 40s (↓ 77-105%; d=.16-.24; p<.01) |
|  | theta | | (d=.13; p=.32) | theta | | (d=.14; p=.32) |
|  | alpha | | ND (d=.03-.10;p=.32-/69) | alpha | | ND (d=.02-.14;p=.24-.92) |
|  | beta | | (d=.04; p=.25) | beta | | (d=.15; p=.05) |
|  | low gamma | | (d=.09; p=.16) | low gamma | | (d=.04; p=.6) |
|  | high gamma | | (d=.03; p=.35) | high gamma | | (d=.05; p=.3) |
|  | low ripple | | (d=.18; p=.08) | low ripple | | (d=.12; p=.23) |
|  | high ripple | | -40, -35s (↓ 80-93%; d=.11-.17;p<.01) | high ripple | | 230s (↑ 27-33%; d=.02-.13; p<.01) |
| Superior parietal lobule | delta | | (d=.55; p=.01) | delta | | 45s (↓ 56-161%; d=.53-.55; p<.001) |
|  | theta | | (d=.09; p=.05) | theta | | (d=.14; p=.03) |
|  | alpha | | ND (d=.03-.10;p=.65-1) | alpha | | ND (d=.05-.17; p=.53-/79) |
|  | beta | | (d=.33; p=.02) | beta | | (d=.26; p=.05) |
|  | low gamma | | (d=.17; p=.01) | low gamma | | (d=.19; p=.11) |
|  | high gamma | | (d=.03; p=.5) | high gamma | | (d=.09; p=.18) |
|  | low ripple | | (d=.17; p=.05) | low ripple | | (d=.13; p=.07) |
|  | high ripple | | -215s (↑ 21-51%; d=.34-.74; p<.001) | high ripple | | 50s (↑ 23-44%; d=.09-.66; p<.001) |
| Middle and inferior temporal gyrus, temporal pole, and planum polare | delta | | ND (d=.05-.34;p=.13-.68) | delta | | 20s (↓ 44-69%; d=.42-.53; p<.001) |
|  | theta | | (d=.07; p=.35) | theta | | (d=.15; p=.21) |
|  | alpha | | ND (d=.03-.14;p=.36-.87) | alpha | | ND (d=.05-.12;p=.12-1) |
|  | beta | | (d=.27; p=.08) | beta | | (d=.24; p=.10) |
|  | low gamma | | (d=.16; p=.10) | low gamma | | 50s (↑ 6-14%; d=.14-.18;p<.001 |
|  | high gamma | | (d=.05; p=.47) | high gamma | | (d=.13; p=.25) |
|  | low ripple | | (d=.16; p=.06) | low ripple | | 50s (↑ 18-28%; d=.11-.26;p<.01) |
|  | high ripple | | -235s (↓ 21-24%; d=.24-.48; p<.001) | high ripple | | 50s (↑ 19-24%; d=.06-.23;p<.01) |
|  |  | |  |  | |  |
| Frontal operculum | **Insufficient coverage** | | | | | |
| Medial and basal temporal region | **Insufficient coverage** | | | | | |
| Medial parietal lobe | **Insufficient coverage** | | | | | |
| Superior temporal gyrus | **Insufficient coverage** | | | | | |
| Supplementary motor cortex | **Insufficient coverage** | | | | | |
| Medial and basal temporal region | **Insufficient coverage** | | | | | |
| Medial parietal lobe | **Insufficient coverage** | | | | | |
| Superior temporal gyrus | **Insufficient coverage** | | | | | |
| Supplementary motor cortex | **Insufficient coverage** | | | | | |
| Medial frontal cortex (including medial segment of superior frontal gyrus) | **Insufficient coverage** | | | | | |
| Anterior and middle cingulate gyrus | **Insufficient coverage** | | | | | |
| Medial and basal temporal region | **Insufficient coverage** | | | | | |
| Transverse temporal gyrus and planum temporale | **Insufficient coverage** | | | | | |
| Pre- and postcentral gyri | **Insufficient coverage** | | | | | |

**Table S2. Spectral density results of awakening from NREM sleep.** Reported here are spectral results for all bands which displayed a significant difference between the awakening process to the wakefulness or sleep reference distributions (wRD and sRD respectively). The results are presented as the time in seconds from the intracranial awakening (IA) when compared to the RDs. Times are given for the convergence to the wRD and the divergence from the sRD. The difference between the awakening process and the RD prior to convergence or after the divergence was tested on the channel level for all channels in the region using a paired Wilcoxon test and assessed with Cliff’s d. The magnitude of the difference between the awakening process to the RDs is reported as relative deviation, which is the change in percentage compared to the RDs. The direction of change is represented as ↑↓ if the time after the awakening corresponded to an increase ↑ or decrease ↓ when compared to the reference distribution. Note: non significant results are reported with the median effect size and p value throughout the duration. Two wakefulness baselines were utilized: one from the prior evening and one from the prior morning. Any differences between these baselines, presented in this order, are detailed in the Table.

**Table S3**

| Network | NREM | | | | | |
| --- | --- | --- | --- | --- | --- | --- |
|  | Relative deviation from wRD | | | | Relative deviation from sRD | |
| Somatomotor | | delta | (d=.28; p=.12) | delta | | (d=.31; p=.1) |
|  |  | theta | (d=.07; p=.22) | theta | | (d=.04; p=.25) |
|  |  | alpha | (d=.02; p=.62) | alpha | | (d=.02; p=.68) |
|  |  | beta | (d=.23; p=.1) | beta | | (d=.22; p=.12) |
|  |  | low gamma | (d=.12; p=.15) | low gamma | | (d=.08; p=.05) |
|  |  | high gamma | (d=.03; p=.43) | high gamma | | (d=.51; p=.08) |
|  |  | low ripple | (d=.29; p=.23) | low ripple | | (d=.16; p=.05) |
|  |  | high ripple | -20s (↓ 119-124%; d=.83-.85; p<.001) | high ripple | | 230s (↑ 20-29%; d=.15-.78; p<.001) |
| Somatomotor -Ventral attention | | delta | (d=.05; p=.46) | delta | | (d=.33; p=.08) |
|  |  | theta | (d=.07; p=.22) | theta | | (d=.05; p=.53) |
|  |  | alpha | (d=.53; p=.15) | alpha | | (d=.03; p=.72) |
|  |  | beta | (d=.05; p=.44) | beta | | (d=.12; p=.02) |
|  |  | low gamma | (d=.04; p=.62) | low gamma | | (d=.06; p=.29) |
|  |  | high gamma | (d=.19; p=.32) | high gamma | | (d=.62; p=.13) |
|  |  | low ripple | (d=.05; p=.12) | low ripple | | (d=.12; p=.06) |
|  |  | high ripple | -40s (↓ 153-188%; d=.65-.68; p<.001) | high ripple | | 255s (↑ 20-28%; d=.25-.82; p<.001) |
| Somatomotor -Frontoparietal | | delta | (d=.33; p=.06) | delta | | (d=.51; p=.1) |
|  |  | theta | (d=.19; p=.07) | theta | | (d=.1; p=.41) |
|  |  | alpha | (d=.11; p=.33) | alpha | | (d=.1; p=.42) |
|  |  | beta | (d=.46; p=.1) | beta | | (d=.25; p=.01) |
|  |  | low gamma | (d=.06; p=.72) | low gamma | | (d=.08; p=.59) |
|  |  | high gamma | (d=.11; p=.34) | high gamma | | (d=.16; p=.09) |
|  |  | low ripple | (d=.23; p=.05) | low ripple | | (d=.14; p=.17) |
|  |  | high ripple | (d=.09; p=.48) | high ripple | | 370s (↑ 20-44%; d=.20-.66; p<.001) |
| Somatomotor - Default | | delta | (d=.32; p=.15) | delta | | (d=.09; p=.48) |
|  |  | theta | (d=.12; p=.39) | theta | | (d=.43; p=.09) |
|  |  | alpha | (d=.12; p=.44) | alpha | | (d=.02; p=.94) |
|  |  | beta | (d=.71; p=.1) | beta | | (d=.16; p=.25) |
|  |  | low gamma | (d=.07; p=.74) | low gamma | | (d=.25; p=.03) |
|  |  | high gamma | (d=.06; p=.76) | high gamma | | (d=.06; p=.78) |
|  |  | low ripple | (d=.03; p=.93) | low ripple | | (d=.55; p=.1) |
|  |  | high ripple | -20, -25s (↓ 229-288%; d=.59-.72; p<.001) | high ripple | | 390s (↑ 16-35%; d=.35-.76; p<.001) |
| Dorsal attention | | delta | (d=.36; p=.17) | delta | | (d=.17; p=.12) |
|  |  | theta | (d=.05; p=.69) | theta | | (d=.13; p=.08) |
|  |  | alpha | (d=.05; p=.62) | alpha | | (d=.08; p=.35) |
|  |  | beta | (d=.22; p=.1) | beta | | (d=.19; p=.10) |
|  |  | low gamma | (d=.14; p=.06) | low gamma | | (d=.1; p=.19) |
|  |  | high gamma | (d=.03; p=.83) | high gamma | | (d=.23; p=.25) |
|  |  | low ripple | (d=.04; p=.17) | low ripple | | 50s (↑ 18-32%; d=.13-.32; p<.001) |
|  |  | high ripple | -345s (↓ 29-56%; d=.79-.83; p<.001) | high ripple | | 95s (↑ 48-61%; d=.34-.61; p<.001) |
| Dorsal attention - Frontoparietal | | delta | (d=.1; p=.4) | delta | | (d=.21; p=.12) |
|  |  | theta | (d=.05; p=.74) | theta | | (d=.15; p=.15) |
|  |  | alpha | (d=.06; p=.67) | alpha | | (d=.07; p=.61) |
|  |  | beta | (d=.13; p=.12) | beta | | 110s (↓ 9-11%; d=.17-.30; p<.001) |
|  |  | low gamma | (d=.04; p=.83) | low gamma | | (d=.02; p=.93) |
|  |  | high gamma | (d=.13; p=.23) | high gamma | | (d=.14; p=.17) |
|  |  | low ripple | (d=.21; p=.06) | low ripple | | 80s (↑ 25-29%; d=.41-.46; p<.001) |
|  |  | high ripple | (d=.27; p=.05) | high ripple | | 45s (↑ 49-51%; d=.29-.56; p<.001) |
| Dorsal attention - Default | | delta | (d=.22; p=.14) | delta | | (d=.31; p=.02) |
|  |  | theta | (d=.05; p=.86) | theta | | (d=.11; p=.62) |
|  |  | alpha | (d=.07; p=.78) | alpha | | (d=.05; p=.86) |
|  |  | beta | (d=.53; p=.06) | beta | | -95s (↓ 8-27%; d=.13-.33; p<.001) |
|  |  | low gamma | (d=.1; p=.65) | low gamma | | (d=.1; p=.63) |
|  |  | high gamma | (d=.06; p=.84) | high gamma | | (d=.29; p=.06) |
|  |  | low ripple | (d=.17; p=.31) | low ripple | | 50s (↑ 18-23%; d=.26-.39; p<.01) |
|  |  | high ripple | (d=.05; p=.86) | high ripple | | (d=.39; p=.11) |
| Default | | delta | (d=.02; p=.97) | delta | | (d=.06; p=.91) |
|  |  | theta | (d=.02; p=.97) | theta | | (d=.04; p=.94) |
|  |  | alpha | (d=.04; p=.94) | alpha | | (d=.01; p=.99) |
|  |  | beta | (d=.1; p=.83) | beta | | (d=.07; p=.88) |
|  |  | low gamma | (d=.12; p=.75) | low gamma | | (d=.11; p=.8) |
|  |  | high gamma | (d=.09; p=.84) | high gamma | | (d=.04; p=.94) |
|  |  | low ripple | (d=.07; p=.29) | low ripple | | 220s (↑ 12-17%; d=.15-.17; p<.001) |
|  |  | high ripple | (d=.17; p=.19) | high ripple | | 200s (↑ 50-59%; d=.23-.35; p<.001) |
| Default - Ventral attention | | delta | (d=.15; p=.38) | delta | | (d=.22; p=.16) |
|  |  | theta | (d=.05; p=.86) | theta | | (d=.06; p=.83) |
|  |  | alpha | (d=.08; p=.74) | alpha | | (d=.14; p=.47) |
|  |  | beta | (d=.35; p=.10) | beta | | (d=.18; p=.27) |
|  |  | low gamma | (d=.04; p=.9) | low gamma | | (d=.1; p=.64) |
|  |  | high gamma | (d=.02; p=.95) | high gamma | | (d=.3; p=.5 |
|  |  | low ripple | (d=.12; p=.55) | low ripple | | 350s (↑ 12-24%; d=.20-.22; p<.01) |
|  |  | high ripple | -40s (↓ 125-147%; d=.10-.22; p<.01) | high ripple | | 240s (↑ 18-22%; d=.11-.16; p<.001) |
| Default - Frontoparietal | | delta | (d=.02; p=.93) | delta | | (d=.05; p=.81) |
|  |  | theta | (d=.02; p=.94) | theta | | (d=.07; p=.74) |
|  |  | alpha | (d=.08; p=.67) | alpha | | (d=.03; p=.9) |
|  |  | beta | (d=.09; p=.56) | beta | | 55s (↓ 3-20%; d=.07-.13; p<.001) |
|  |  | low gamma | (d=.03; p=.9) | low gamma | | (d=.07; p=.74) |
|  |  | high gamma | (d=.09; p=.55) | high gamma | | (d=.06; p=.84) |
|  |  | low ripple | (d=.13; p=.35) | low ripple | | (d=.13; p=.33) |
|  |  | high ripple | (d=.21; p=.06) | high ripple | | 380s (↑ 13-18%; d=.53-.54; p<.001) |
| Frontoparietal | | delta | (d=.04; p=.78) | delta | | (d=.02; p=.91) |
|  |  | theta | (d=.02; p=.91) | theta | | (d=.08; p=.35) |
|  |  | alpha | (d=.09; p=.25) | alpha | | (d=.07; p=.43) |
|  |  | beta | (d=.04; p=.77) | beta | | (d=.14; p=.06) |
|  |  | low gamma | (d=.04; p=.79) | low gamma | | (d=.06; p=.59) |
|  |  | high gamma | (d=.14; p=.06) | high gamma | | (d=.06; p=.53) |
|  |  | low ripple | (d=.38; p=.06) | low ripple | | (d=.27; p=.05) |
|  |  | high ripple | (d=.44; p=.05) | high ripple | | 45s (↑ 34-40%; d=.77-.79; p<.001) |
| Ventral Attention - Frontoparietal | | delta | (d=.12; p=.31) | delta | | (d=.2; p=.05) |
|  |  | theta | (d=.07; p=.64) | theta | | (d=.09; p=.53) |
|  |  | alpha | (d=.03; p=.91) | alpha | | (d=.08; p=.61) |
|  |  | beta | (d=.21; p=.03) | beta | | (d=.08; p=.55) |
|  |  | low gamma | (d=.03; p=.87) | low gamma | | (d=.08; p=.6) |
|  |  | high gamma | (d=.03; p=.89) | high gamma | | (d=.02; p=.93) |
|  |  | low ripple | (d=.07; p=.63) | low ripple | | (d=.18; p=.07) |
|  |  | high ripple | (d=.04; p=.86) | high ripple | | (d=.04; p=.84) |
| Visual | | **Insufficient coverage** | | | | |
| Visual - Somatomotor | | **Insufficient coverage** | | | | |
| Visual - Dorsal Attention | | **Insufficient coverage** | | | | |
| Visual - Ventral Attention | | **Insufficient coverage** | | | | |
| Visual - Limbic | | **Insufficient coverage** | | | | |
| Visual - Frontoparietal | | **Insufficient coverage** | | | | |
| Visual - Default mode | | **Insufficient coverage** | | | | |
| Somatomotor - Dorsal Attention | | **Insufficient coverage** | | | | |
| Somatomotor - Limbic | | **Insufficient coverage** | | | | |
| Dorsal Attention - Ventral Attention | | **Insufficient coverage** | | | | |
| Dorsal Attention - Limbic | | **Insufficient coverage** | | | | |
| Ventral Attention - Limbic | | **Insufficient coverage** | | | | |
| Limbic - Frontoparietal | | **Insufficient coverage** | | | | |
| Limbic - Frontoparietal | | **Insufficient coverage** | | | | |
| Limbic - Default mode | | **Insufficient coverage** | | | | |

**Table S3. Phase connectivity results of awakening from NREM.** Reported here are phase locking value (PLV) results for all bands which displayed a significant difference between the awakening process to the wakefulness or sleep reference distributions (wRD and sRD respectively). The results are presented as the time in seconds from the intracranial awakening (IA) when compared to the RDs. Times are given for the convergence to the wRD and the divergence from the sRD. The difference between the awakening process and the RD prior to convergence or after the divergence was tested on the channel level for all channels-pairs within a network or between two different networks, using a paired Wilcoxon test and assessed with Cliff’s d. The magnitude of the difference between the awakening process to the RDs is reported as relative deviation, which is the change in percentage compared to the RDs. The direction of change is represented as ↑↓ if the time after the awakening corresponded to an increase ↑ or decrease ↓ when compared to the reference distribution. ND- never diverged from the RD in any frequency band. Note: non significant results are reported with the median effect size and p value throughout the duration. Two wakefulness baselines were utilized: one from the prior evening and one from the prior morning. Any differences between these baselines, presented in this order, are detailed in the Table.

**Table S4**

| Region | REM | | | | |
| --- | --- | --- | --- | --- | --- |
|  | wRD | | sRD | | |
| Anterior and middle cingulate gyrus | delta | (d=.33; p=.06) | | delta | (d=.18; p=.05) |
|  | theta | (d=.17; p=.08) | | theta | (d=.07; p=.56) |
|  | alpha | (d=.21; p=.08) | | alpha | (d=.09; p=.31) |
|  | beta | (d=.09; p=.41) | | beta | (d=.41; p=.04) |
|  | low gamma | (d=.08; p=.28) | | low gamma | (d=.08; p=.68) |
|  | high gamma | (d=.07; p=.41) | | high gamma | (d=.17; p=.12) |
|  | low ripple | -45, -55s (↓ 41-47%; d=.31-.33; p<.01) | | low ripple | 260s (↑ 5-21%; d=.21-.44; p<.01) |
|  | high ripple | -40s (↓ 36-41%; d=.38-.42; p<.01) | | high ripple | 45s (↑ 5-22%; d=.22-.29; p<.01) |
| Central operculum and opercular part of inferior frontal gyrus | delta | (d=.2; p=.06) | | delta | -350s (↓ 13-87%; d=.11-.73; p<.001) |
|  | theta | (d=.37; p=.10) | | theta | (d=.41; p=.12) |
|  | alpha | (d=.48; p=.12) | | alpha | (d=.35; p=.06) |
|  | beta | (d=.27; p=.10) | | beta | (d=.41; p=.1) |
|  | low gamma | (d=.24; p=.07) | | low gamma | (d=.16; p=.1) |
|  | high gamma | (d=.07; p=.14) | | high gamma | (d=.14; p=.08) |
|  | low ripple | (d=.06; p=.33) | | low ripple | (d=.14; p=.05) |
|  | high ripple | -35s (↓ 59-62%; d=.16-.22; p<.01) | | high ripple | ND |
| Inferior parietal lobule | delta | (d=.17; p=.07) | | delta | (d=.06; p=.6) |
|  | theta | (d=.09; p=.06) | | theta | (d=.06; p=.07) |
|  | alpha | (d=.07; p=.11) | | alpha | (d=.07; p=.13) |
|  | beta | (d=.04; p=.05) | | beta | (d=.02; p=.22) |
|  | low gamma | (d=.05; p=.1) | | low gamma | (d=.04; p=.05) |
|  | high gamma | (d=.04; p=.11) | | high gamma | (d=.07; p=.05) |
|  | low ripple | 45s (↓ 42-47%; d=.11-.27; p<.001) | | low ripple | 165s (↑ 12-15%; d=.03-.15; p<.01) |
|  | high ripple | NC (↓ 40-65%; d=.02-.28; p<.05) | | high ripple | ND |
| Insula | delta | (d=.23; p=.06) | | delta | (d=.04; p=.36) |
|  | theta | (d=.1; p=.05) | | theta | (d=.13; p=.13) |
|  | alpha | (d=.15; p=.13) | | alpha | (d=.09; p=.24) |
|  | beta | (d=.04; p=.33) | | beta | (d=.03; p=.45) |
|  | low gamma | (d=.08; p=.01) | | low gamma | (d=.1; p=.03) |
|  | high gamma | (d=.05; p=.15) | | high gamma | (d=.04; p=.18) |
|  | low ripple | (d=.03; p=.16) | | low ripple | (d=.19; p=.06) |
|  | high ripple | (d=.06; p=.07) | | high ripple | 305s (↑ 5-17%; d=.17-.25; p<.05) |
| Medial and basal temporal region | delta | (d=.36; p=.06) | | delta | -480s (↓ 13-72%; d=.30-.42; p<.001) |
|  | theta | (d=.14; p=.87) | | theta | (d=.1; p=.4) |
|  | alpha | (d=.1; p=.56) | | alpha | (d=.08; p=.46) |
|  | beta | (d=.04; p=.15) | | beta | (d=.12; p=.11) |
|  | low gamma | (d=.16; p=.09) | | low gamma | (d=.04; p=.08) |
|  | high gamma | NC (↓ 52-92%; d=.31-.34; p<.001) | | high gamma | (d=.35; p=.06) |
|  | low ripple | NC (↓ 41-85%; d=.15-.52; p<.001) | | low ripple | 55s (↑ 13-19%; d=.30-.31; p<.001) |
|  | high ripple | NC (↓ 49-88%; d=.35-.57; p<.01) | | high ripple | ND |
| Medial frontal cortex | delta | (d=.3; p=.07) | | delta | (d=.29; p=.06) |
|  | theta | (d=.16; p=.06) | | theta | (d=.2; p=.05) |
|  | alpha | (d=.27; p=.1) | | alpha | (d=.24; p=.11) |
|  | beta | (d=.13; p=.12) | | beta | (d=.26; p=.08) |
|  | low gamma | (d=.07; p=.33) | | low gamma | (d=.09; p=.43) |
|  | high gamma | (d=.05; p=.18) | | high gamma | (d=.04; p=.33) |
|  | low ripple | 45s (↓ 37-40%; d=.18-.19; p<.001) | | low ripple | 35s (↑ 6-25%; d=.18-.30; p<.01) |
|  | high ripple | -40, -35s (↓ 36-43%; d=.18-.19; p<.001) | | high ripple | 45s (↑ 12-26%; d=.09-.12; p<.001) |
| Middle and inferior temporal gyrus, temporal pole, and planum polare | delta | (d=.23; p=.06) | | delta | -405s (↓ 19-58%; d=.23-.43; p<.001) |
|  | theta | (d=.06; p=.09) | | theta | (d=.05; p=.15) |
|  | alpha | (d=.11; p=.1) | | alpha | (d=.16; p=.08) |
|  | beta | (d=.13; p=.6) | | beta | 25s (↑ 15-19%; d=.15-.20; p<.001) |
|  | low gamma | (d=.07; p=.05) | | low gamma | (d=.2; p=.05) |
|  | high gamma | -5s (↓ 46-50%; d=.22-.23; p<.001) | | high gamma | 75s (↑ 9-13%; d=.04-.15; p<.001) |
|  | low ripple | 10s (↓ 81-86%; d=.47-.48; p<.001) | | low ripple | -50s (↑ 18-24%; d=.20-.44; p<.001) |
|  | high ripple | -10s (↓ 54-77%; d=.35-.36; p<.001) | | high ripple | -25s (↑ 22-27%; d=.24-.34; p<.001) |
| Superior, middle, and orbital frontal gyri and anterior part of inferior frontal gyrus | delta | (d=.2; p=.16) | | delta | -480s (↓ 37-87%; d=.16-.57; p<.01) |
|  | theta | (d=.03; p=.13) | | theta | (d=.11; p=.08) |
|  | alpha | (d=.04; p=.11) | | alpha | (d=.07; p=.1) |
|  | beta | (d=.14; p=.19) | | beta | (d=.05; p=.26) |
|  | low gamma | (d=.1; p=.12) | | low gamma | (d=.09; p=.08) |
|  | high gamma | (d=.06; p=.06) | | high gamma | (d=.1; p=.06) |
|  | low ripple | 0s (↓ 39-44%; d=.16-.19; p<.001) | | low ripple | 55s (↑ 10-29%; d=.11-.36; p<.001) |
|  | high ripple | 0s (↓ 39-42%; d=.12-.14; p<.001) | | high ripple | 50s (↑ 14-31%; d=.08-.2; p<.001) |
| Superior temporal gyrus | delta | (d=.16; p=.10) | | delta | (d=.09; p=.18) |
|  | theta | (d=.17; p=.12) | | theta | (d=.15; p=.05) |
|  | alpha | (d=.11; p=.08) | | alpha | 50s (↑ 23-40%; d=.14-.42; p<.001) |
|  | beta | (d=.1; p=.06) | | beta | -40s (↑ 11-24%; d=.11-.24; p<.001) |
|  | low gamma | (d=.1; p=.05) | | low gamma | (d=.11; p=.15) |
|  | high gamma | (d=.02; p=.33) | | high gamma | (d=.05; p=.15) |
|  | low ripple | -30s (↓ 43-47%; d=.25-.27; p<.001) | | low ripple | 55s (↑ 19-36%; d=.21-.44; p<.01) |
|  | high ripple | (d=.08; p=.09) | | high ripple | -20s (↑ 12-39%; d=.09-.27; p<.001) |
| Medial parietal lobe | delta | (d=.22; p=.09) | | delta | (d=.13; p=.21) |
|  | theta | (d=.17; p=.1) | | theta | (d=.09; p=.12) |
|  | alpha | (d=.16; p=.06) | | alpha | (d=.09; p=.07) |
|  | beta | (d=.08; p=.12) | | beta | (d=.08; p=.25) |
|  | low gamma | (d=.08; p=.05) | | low gamma | (d=.13; p=.25) |
|  | high gamma | (d=.06; p=.15) | | high gamma | (d=.09; p=.5) |
|  | low ripple | (d=.12; p=.07) | | low ripple | (d=.12; p=.15) |
|  | high ripple | (d=.1; p=.25) | | high ripple | (d=.08; p=.64) |
| Superior parietal lobule | delta | (d=.38; p=.07) | | delta | (d=.38; p=.06) |
|  | theta | (d=.36; p=.05) | | theta | (d=.36; p=.07) |
|  | alpha | (d=.31; p=.05) | | alpha | (d=.19; p=.06) |
|  | beta | (d=.16; p=.08) | | beta | (d=.16; p=.06) |
|  | low gamma | (d=.11; p=.09) | | low gamma | (d=.21; p=.08) |
|  | high gamma | (d=.14; p=.13) | | high gamma | (d=.14; p=.08) |
|  | low ripple | (d=.11; p=.1) | | low ripple | (d=.04; p=.53) |
|  | high ripple | (d=.11; p=.13) | | high ripple | (d=.06; p=.6) |
| Medial frontal cortex (including medial segment of superior frontal gyrus) | delta | (d=.3; p=.06) | | delta | (d=.29; p=.10) |
|  | theta | (d=.16; p=.08) | | theta | (d=.2; p=.10) |
|  | alpha | (d=.27; p=.1) | | alpha | (d=.24; p=.12) |
|  | beta | (d=.13; p=.12) | | beta | (d=.26; p=.08) |
|  | low gamma | (d=.07; p=.33) | | low gamma | (d=.09; p=.43) |
|  | high gamma | (d=.05; p=.18) | | high gamma | (d=.04; p=.33) |
|  | low ripple | (d=.21; p=.06) | | low ripple | (d=.26; p=.06) |
|  | high ripple | (d=.15; p=.33) | | high ripple | (d=.22; p=.05) |
| Frontal operculum | delta | (d=.07; p=.53) | | delta | (d=.21; p=.81) |
|  | theta | (d=.05; p=.53) | | theta | (d=.11; p=.81) |
|  | alpha | (d=.06; p=.6) | | alpha | (d=.28; p=.94) |
|  | beta | (d=.11; p=.6) | | beta | (d=.23; p=.08) |
|  | low gamma | (d=.1; p=.6) | | low gamma | (d=.16; p=.2) |
|  | high gamma | (d=.07; p=.29) | | high gamma | (d=.16; p=.08) |
|  | low ripple | (d=.11; p=.09) | | low ripple | (d=.31; p=.05) |
|  | high ripple | (d=.16; p=.06) | | high ripple | (d=.46; p=.06) |
| Transverse temporal gyrus and planum temporale | delta | (d=.21; p=.16) | | delta | (d=.11; p=.67) |
|  | theta | (d=.11; p=.24) | | theta | (d=.19; p=.29) |
|  | alpha | (d=.06; p=.53) | | alpha | (d=.09; p=.46) |
|  | beta | (d=.07; p=.35) | | beta | (d=.09; p=.53) |
|  | low gamma | (d=.04; p=.74) | | low gamma | (d=.11; p=.24) |
|  | high gamma | (d=.06; p=.67) | | high gamma | (d=.21; p=.08) |
|  | low ripple | (d=.02; p=.88) | | low ripple | (d=.21; p=.05) |
|  | high ripple | (d=.09; p=.65) | | high ripple | (d=.23; p=.08) |
| Medial occipital lobe | **Insufficient coverage** | | | | |
| Lateral occipital lobe | **Insufficient coverage** | | | | |
| Pre- and postcentral gyri | **Insufficient coverage** | | | | |
| Supplementary motor cortex | **Insufficient coverage** | | | | |

**Table S4. Spectral density results of awakening from REM sleep.** Reported here are spectral results for all bands which displayed a significant difference between the awakening process to the wakefulness or sleep reference distributions (wRD and sRD respectively). The results are presented as the time in seconds from the intracranial awakening (IA) when compared to the RDs. Times are given for the convergence to the wRD and the divergence from the sRD. The difference between the awakening process and the RD prior to convergence or after the divergence was tested on the channel level for all channels in the region using a paired Wilcoxon test and assessed with Cliff’s d. The magnitude of the difference between the awakening process to the RDs is reported as relative deviation, which is the change in percentage compared to the RDs. The direction of change is represented as ↑↓ if the time after the awakening corresponded to an increase ↑ or decrease ↓ when compared to the reference distribution. ND- never diverged from the RD in any frequency band, NC- never converged back the RD. Note that while the medial and basal temporal region never significantly converged to the wRD they did show an upward trend. Note: non significant results are reported with the median effect size and p value throughout the duration. Two wakefulness baselines were utilized: one from the prior evening and one from the prior morning. Any differences between these baselines, presented in this order, are detailed in the Table.

**Table S5**

| Network | REM | | | |
| --- | --- | --- | --- | --- |
|  | wRD | | sRD | |
| Limbic | delta | (d=.04; p=.52) | delta | (d=.03; p=.7) |
|  | theta | (d=.06; p=.28) | theta | (d=.04; p=.55) |
|  | alpha | (d=.04; p=.59) | alpha | (d=.06; p=.34) |
|  | beta | (d=.03; p=.65) | beta | (d=.08; p=.17) |
|  | low gamma | (d=.05; p=.41) | low gamma | (d=.05; p=.41) |
|  | high gamma | (d=.01; p=.88) | high gamma | (d=.13; p=.06) |
|  | low ripple | -40s (↓ 36-40%; d=.30-.38; p<.001) | low ripple | 50s (↑ 28-39%; d=.30-.35; p<.001) |
|  | high ripple | (d=.01; p=.91) | high ripple | (d=.23; p=.06) |
| Limbic - Ventral attention | delta | (d=.05; p=.39) | delta | (d=.06; p=.26) |
|  | theta | (d=.03; p=.59) | theta | (d=.05; p=.36) |
|  | alpha | (d=.09; p=.09) | alpha | (d=.09; p=.08) |
|  | beta | (d=.04; p=.46) | beta | (d=.03; p=.62) |
|  | low gamma | (d=.07; p=.19) | low gamma | (d=.09; p=.1) |
|  | high gamma | (d=.01; p=.84) | high gamma | (d=.08; p=.13) |
|  | low ripple | (d=.17; p=.10) | low ripple | 50s (↑ 21-24%; d=.34-.44; p<.001) |
|  | high ripple | (d=.03; p=.59) | high ripple | (d=.15; p=.05) |
| Limbic - Visual | delta | (d=.11; p=.23) | delta | (d=.17; p=.07) |
|  | theta | (d=.1; p=.28) | theta | (d=.15; p=.12) |
|  | alpha | (d=.17; p=.06) | alpha | (d=.15; p=.11) |
|  | beta | (d=.1; p=.32) | beta | (d=.06; p=.55) |
|  | low gamma | (d=.06; p=.57) | low gamma | (d=.1; p=.32) |
|  | high gamma | (d=.06; p=.54) | high gamma | (d=.09; p=.39) |
|  | low ripple | (d=.14; p=.13) | low ripple | (d=.24; p=.05) |
|  | high ripple | NC (↑ 21-24%; d=.21-.35; p<.001) | high ripple | 230s (↓ 6-7%; d=.20-.23; p<.01) |
| Limbic - Somatomotor | delta | (d=.16; p=.08) | delta | (d=.08; p=.25) |
|  | theta | (d=.06; p=.38) | theta | (d=.1; p=.12) |
|  | alpha | (d=.1; p=.14) | alpha | (d=.15; p=.02) |
|  | beta | (d=.1; p=.12) | beta | (d=.11; p=.09) |
|  | low gamma | (d=.07; p=.29) | low gamma | (d=.15; p=.01) |
|  | high gamma | (d=.04; p=.65) | high gamma | (d=.33; p=.06) |
|  | low ripple | (d=.14; p=.17) | low ripple | 45s (↑ 22-23%; d=.31-.41; p<.001) |
|  | high ripple | -455, -450s (↑ 15-16%; d=.51-54; p<.001) | high ripple | (d=.51; p=.05) |
| Somatomotor - Ventral attention | delta | (d=.15; p=.18) | delta | (d=.1; p=.10) |
|  | theta | (d=.11; p=.14) | theta | (d=.12; p=.25) |
|  | alpha | (d=.12; p=0) | alpha | (d=.11; p=.17) |
|  | beta | (d=.08; p=.09) | beta | (d=.1; p=.06) |
|  | low gamma | (d=.04; p=.43) | low gamma | (d=.03; p=.53) |
|  | high gamma | (d=.04; p=.42) | high gamma | (d=.46; p=.07) |
|  | low ripple | (d=.14; p=.06) | low ripple | 45s (↑ 21-22%; d=.14-.38; p<.01) |
|  | high ripple | -75s (↑ 14-42%; d=.62-.65; p<.001) | high ripple | (d=.65; p=.05) |
| Limbic - Default | delta | (d=.12; p=.13) | delta | (d=.07; p=.07) |
|  | theta | (d=.02; p=.6) | theta | (d=.03; p=.41) |
|  | alpha | (d=.03; p=.41) | alpha | (d=.05; p=.19) |
|  | beta | (d=.03; p=.39) | beta | (d=.03; p=.39) |
|  | low gamma | (d=.06; p=.09) | low gamma | (d=.06; p=.07) |
|  | high gamma | (d=.01; p=.77) | high gamma | (d=.07; p=.05) |
|  | low ripple | (d=.11; p=.07) | low ripple | 45s (↑ 20-24%; d=.35-.41; p<.001) |
|  | high ripple | (d=0; p=.92) | high ripple | 390s (↑ 14-19%; d=.02-.29; p<.001) |
| Limbic - Frontoparietal | delta | (d=.12; p=.07) | delta | (d=.14; p=.12) |
|  | theta | (d=.03; p=.48) | theta | (d=.04; p=.38) |
|  | alpha | (d=.1; p=.05) | alpha | (d=.11; p=.09) |
|  | beta | (d=.04; p=.36) | beta | (d=.08; p=.05) |
|  | low gamma | (d=.06; p=.13) | low gamma | (d=.12; p=0) |
|  | high gamma | (d=.02; p=.62) | high gamma | (d=.07; p=.06) |
|  | low ripple | (d=.13; p=.05) | low ripple | 45s (↑ 28-39%; d=.30-.38; p<.001) |
|  | high ripple | (d=.02; p=.67) | high ripple | (d=.08; p=.05) |
| Default | delta | (d=.1; p=.12) | delta | (d=.11; p=.08) |
|  | theta | (d=.03; p=.16) | theta | (d=.05; p=.13) |
|  | alpha | (d=.08; p=.06) | alpha | (d=.07; p=.07) |
|  | beta | (d=.03; p=.21) | beta | (d=.03; p=.12) |
|  | low gamma | (d=.04; p=.06) | low gamma | (d=.06; p=.05) |
|  | high gamma | (d=.01; p=.71) | high gamma | (d=.06; p=.21) |
|  | low ripple | (d=.12; p=.05) | low ripple | 45s (↑ 18-34%; d=.27-.51; p<.001) |
|  | high ripple | (d=.15; p=.14) | high ripple | 440s (↑ 11-14%; d=.05-.21; p<.001) |
| Default- Dorsal attention | delta | (d=.18; p=.08) | delta | (d=.09; p=.11) |
|  | theta | (d=.1; p=.12) | theta | (d=.19; p=.12) |
|  | alpha | (d=.09; p=.09) | alpha | (d=.08; p=.09) |
|  | beta | (d=.07; p=.03) | beta | (d=.06; p=.08) |
|  | low gamma | (d=.09; p=.05) | low gamma | (d=.1; p=.06) |
|  | high gamma | (d=.04; p=.33) | high gamma | (d=.21; p=.08) |
|  | low ripple | (d=.18; p=.05) | low ripple | 50s (↑ 9-16%; d=.37-.67; p<.01) |
|  | high ripple | (d=.05; p=.13) | high ripple | (d=.17; p=.05) |
| Default - Ventral attention | delta | (d=.09; p=.25) | delta | (d=.1; p=.32) |
|  | theta | (d=.06; p=.15) | theta | (d=.07; p=.22) |
|  | alpha | (d=.08; p=.16) | alpha | (d=.08; p=.11) |
|  | beta | (d=.01; p=.8) | beta | (d=.01; p=.85) |
|  | low gamma | (d=.1; p=.10) | low gamma | (d=.07; p=.12) |
|  | high gamma | (d=.03; p=.45) | high gamma | (d=.08; p=.08) |
|  | low ripple | (d=.15; p=.05) | low ripple | 50s (↑ 14-19%; d=.27-.41; p<.01) |
|  | high ripple | (d=.03; p=.31) | high ripple | (d=.2; p=.05) |
| Default - Somatomotor | delta | (d=.14; p=.12) | delta | (d=.05; p=.11) |
|  | theta | (d=.12; p=.08) | theta | (d=.1; p=.07) |
|  | alpha | (d=.13; p=.22) | alpha | (d=.11; p=.18) |
|  | beta | (d=.03; p=.47) | beta | (d=.04; p=.24) |
|  | low gamma | (d=.11; p=.13) | low gamma | (d=.08; p=.09) |
|  | high gamma | (d=.02; p=.56) | high gamma | (d=.35; p=.24) |
|  | low ripple | (d=.11; p=.06) | low ripple | 50s (↑ 18-28%; d=.30-.45; p<.001) |
|  | high ripple | (d=.06; p=.08) | high ripple | 80s (↓ 8-17%; d=.09-.21; p<.001) |
| Default - Visual | delta | (d=.1; p=.09) | delta | (d=.06; p=.21) |
|  | theta | (d=.05; p=.31) | theta | (d=.09; p=.08) |
|  | alpha | (d=.16; p=.22) | alpha | (d=.05; p=.36) |
|  | beta | (d=.12; p=.11) | beta | (d=.06; p=.29) |
|  | low gamma | (d=.05; p=.38) | low gamma | (d=.05; p=.32) |
|  | high gamma | (d=.04; p=.51) | high gamma | (d=.13; p=.07) |
|  | low ripple | (d=.15; p=.05) | low ripple | 430s (↑ 19-31%; d=.51-.78; p<.01) |
|  | high ripple | (d=.07; p=.18) | high ripple | (d=.38; p=.25) |
| Default - Frontoparietal | delta | (d=.13; p=.24) | delta | (d=.12; p=.22) |
|  | theta | (d=.08; p=.27) | theta | (d=.04; p=.08) |
|  | alpha | (d=.19; p=.18) | alpha | (d=.17; p=.13) |
|  | beta | (d=.07; p=.15) | beta | (d=.05; p=.13) |
|  | low gamma | (d=.06; p=.06) | low gamma | (d=.05; p=.05) |
|  | high gamma | (d=.06; p=.18) | high gamma | (d=.04; p=.06) |
|  | low ripple | (d=.11; p=0) | low ripple | 275s (↑ 13-23%; d=.27-.39; p<.001) |
|  | high ripple | (d=.02; p=.19) | high ripple | (d=.05; p=.06) |
| Dorsal attention | delta | (d=.22; p=.08) | delta | (d=.2; p=.18) |
|  | theta | (d=.1; p=.15) | theta | (d=.19; p=.26) |
|  | alpha | (d=.13; p=.39) | alpha | (d=.07; p=.27) |
|  | beta | (d=.1; p=.01) | beta | (d=.08; p=.25) |
|  | low gamma | (d=.06; p=.17) | low gamma | (d=.08; p=.05) |
|  | high gamma | (d=.04; p=.42) | high gamma | (d=.37; p=.07) |
|  | low ripple | (d=.26; p=.06) | low ripple | 280s (↑ 22-25%; d=.38-.60; p<.001) |
|  | high ripple | (d=.05; p=.23) | high ripple | (d=.27; p=.12) |
| Dorsal attention - Limbic | delta | (d=.13; p=.14) | delta | (d=.09; p=.21) |
|  | theta | (d=.14; p=.08) | theta | (d=.15; p=.12) |
|  | alpha | (d=.12; p=.06) | alpha | (d=.07; p=.3) |
|  | beta | (d=.04; p=.59) | beta | (d=.09; p=.17) |
|  | low gamma | (d=.09; p=.19) | low gamma | (d=.07; p=.3) |
|  | high gamma | (d=.06; p=.44) | high gamma | (d=.14; p=.03) |
|  | low ripple | (d=.06; p=.08) | low ripple | 80s (↑ 31-56%; d=.35-37; p<.001) |
|  | high ripple | (d=.04; p=.61) | high ripple | (d=.29; p=.25) |
| Dorsal attention - Ventral attention | delta | (d=.08; p=.15) | delta | (d=.09; p=.22) |
|  | theta | (d=.07; p=.28) | theta | (d=.18; p=.11) |
|  | alpha | (d=.08; p=.14) | alpha | (d=.1; p=.22) |
|  | beta | (d=.11; p=.31) | beta | (d=.14; p=.13) |
|  | low gamma | (d=.02; p=.68) | low gamma | (d=.05; p=.24) |
|  | high gamma | (d=.08; p=.06) | high gamma | (d=.37; p=.05) |
|  | low ripple | (d=.14; p=.07) | low ripple | 345s (↑ 10-23%; d=.23-53; p<.001) |
|  | high ripple | (d=.06; p=.14) | high ripple | (d=.55; p=.08) |
| Dorsal attention - Frontoparietal | delta | (d=.2; p=.23) | delta | (d=.1; p=.21) |
|  | theta | (d=.05; p=.25) | theta | (d=.19; p=.13) |
|  | alpha | (d=.11; p=.34) | alpha | (d=.06; p=.13) |
|  | beta | (d=.07; p=.07) | beta | (d=.09; p=.08) |
|  | low gamma | (d=.11; p=.09) | low gamma | (d=.07; p=.07) |
|  | high gamma | (d=.05; p=.25) | high gamma | (d=.4; p=.10) |
|  | low ripple | (d=.2; p=.06) | low ripple | 45s (↑ 14-49%; d=.27-51; p<.001) |
|  | high ripple | (d=.03; p=.42) | high ripple | (d=.31; p=.05) |
| Visual | delta | (d=.11; p=.42) | delta | (d=.12; p=.18) |
|  | theta | (d=.06; p=.51) | theta | (d=.04; p=.72) |
|  | alpha | (d=.17; p=.05) | alpha | (d=.08; p=.4) |
|  | beta | (d=.07; p=.47) | beta | (d=.07; p=.47) |
|  | low gamma | (d=.04; p=.71) | low gamma | (d=.04; p=.72) |
|  | high gamma | (d=.04; p=.77) | high gamma | (d=.3; p=.38) |
|  | low ripple | (d=.07; p=.47) | low ripple | (d=.14; p=.11) |
|  | high ripple | -70s (↓ 20-40%; d=.59-.61; p<.001) | high ripple | (d=.62; p=.07) |
| Visual - Somatomotor | delta | (d=.14; p=.22) | delta | (d=.07; p=.27) |
|  | theta | (d=.12; p=.34) | theta | (d=.16; p=.25) |
|  | alpha | (d=.25; p=.10) | alpha | (d=.17; p=.19) |
|  | beta | (d=.12; p=.13) | beta | (d=.05; p=.41) |
|  | low gamma | (d=.08; p=.22) | low gamma | (d=.05; p=.42) |
|  | high gamma | (d=.05; p=.5) | high gamma | (d=.07; p=.42) |
|  | low ripple | (d=.1; p=.10) | low ripple | 225s (↑ 24-39%; d=.31-.51; p<.01) |
|  | high ripple | NC (↑ 75-81%; d=.92-.95; p<.001) | high ripple | (d=.94; p=.05) |
| Somatomotor | delta | (d=.13; p=.13) | delta | (d=.06; p=.11) |
|  | theta | (d=.06; p=.12) | theta | (d=.08; p=.14) |
|  | alpha | (d=.11; p=.23) | alpha | (d=.05; p=.21) |
|  | beta | (d=0; p=.94) | beta | (d=.02; p=.7) |
|  | low gamma | (d=.04; p=.32) | low gamma | (d=.08; p=.05) |
|  | high gamma | (d=.01; p=.8) | high gamma | (d=.51; p=.09) |
|  | low ripple | (d=.14; p=.07) | low ripple | 50s (↑ 14-21%; d=.20-.47; p<.001) |
|  | high ripple | (d=.06; p=.1) | high ripple | (d=.72; p=.06) |
| Somatomotor- Dorsal attention | delta | (d=.12; p=.16) | delta | (d=.06; p=.12) |
|  | theta | (d=.11; p=.10) | theta | (d=.15; p=.13) |
|  | alpha | (d=.15; p=.25) | alpha | (d=.07; p=.09) |
|  | beta | (d=.11; p=.30) | beta | (d=.1; p=.11) |
|  | low gamma | (d=.06; p=.15) | low gamma | (d=.1; p=.09) |
|  | high gamma | (d=.03; p=.44) | high gamma | (d=.67; p=.05) |
|  | low ripple | (d=.3; p=.08) | low ripple | 275s (↑ 23-41%; d=.44-.77; p<.001) |
|  | high ripple | (d=.04; p=.31) | high ripple | (d=.79; p=.05) |
| Somatomotor - Frontoparietal | delta | (d=.24; p=.07) | delta | (d=.12; p=.11) |
|  | theta | (d=.1; p=.23) | theta | (d=.15; p=.15) |
|  | alpha | (d=.12; p=.20) | alpha | (d=.13; p=.42) |
|  | beta | (d=.02; p=.56) | beta | (d=.05; p=.21) |
|  | low gamma | (d=.03; p=.52) | low gamma | (d=.02; p=.72) |
|  | high gamma | (d=.02; p=.6) | high gamma | (d=.48; p=.15) |
|  | low ripple | (d=.09; p=.05) | low ripple | 50s (↑ 18-35%; d=.26-.49; p<.001) |
|  | high ripple | (d=.07; p=.05) | high ripple | 485s (↓ 2-14%; d=.07-.13; p<.01) |
| Ventral attention - Frontoparietal | delta | (d=.12; p=.12) | delta | (d=.08; p=.02) |
|  | theta | (d=.09; p=.11) | theta | (d=.16; p=.13) |
|  | alpha | (d=.1; p=.23) | alpha | (d=.09; p=.17) |
|  | beta | (d=.05; p=.15) | beta | (d=.06; p=.09) |
|  | low gamma | (d=.05; p=.13) | low gamma | (d=.05; p=.15) |
|  | high gamma | (d=.02; p=.68) | high gamma | (d=.23; p=.26) |
|  | low ripple | (d=.14; p=.07) | low ripple | 245s (↑ 4-15%; d=.15-.33; p<.001) |
|  | high ripple | (d=.04; p=.22) | high ripple | (d=.28; p=.18) |
| Frontoparietal | delta | (d=.06; p=.06) | delta | (d=.16; p=.09) |
|  | theta | (d=.1; p=.37) | theta | (d=.07; p=.13) |
|  | alpha | (d=.13; p=.24) | alpha | (d=.13; p=.18) |
|  | beta | (d=.02; p=.36) | beta | (d=.04; p=.09) |
|  | low gamma | (d=.03; p=.30) | low gamma | (d=.03; p=.34) |
|  | high gamma | (d=.02; p=.40) | high gamma | (d=.06; p=.06) |
|  | low ripple | (d=.07; p=.07) | low ripple | 380s (↑ 9-27%; d=.27-.39; p<.001) |
|  | high ripple | (d=.03; p=.18) | high ripple | (d=.06; p=.05) |
| Ventral Attention - Visual | delta | (d=.14; p=.11) | delta | (d=.15; p=.09) |
|  | theta | (d=.07; p=.24) | theta | (d=.13; p=.09) |
|  | alpha | (d=.17; p=.34) | alpha | (d=.09; p=.14) |
|  | beta | (d=.1; p=.08) | beta | (d=.08; p=.23) |
|  | low gamma | (d=.05; p=.48) | low gamma | (d=.04; p=.54) |
|  | high gamma | (d=.07; p=.29) | high gamma | (d=.34; p=.13) |
|  | low ripple | (d=.08; p=.17) | low ripple | (d=.16; p=.12) |
|  | high ripple | (d=.09; p=.16) | high ripple | (d=.59; p=.06) |
| Somatomotor—Default | delta | (d=.14; p=.23) | delta | (d=.05; p=.11) |
|  | theta | (d=.12; p=.33) | theta | (d=.1; p=.19) |
|  | alpha | (d=.13; p=.20) | alpha | (d=.11; p=.23) |
|  | beta | (d=.03; p=.47) | beta | (d=.04; p=.24) |
|  | low gamma | (d=.11; p=.37) | low gamma | (d=.08; p=.11) |
|  | high gamma | (d=.02; p=.56) | high gamma | (d=.35; p=.15) |
|  | low ripple | (d=.11; p=.06) | low ripple | (d=.29; p=.06) |
|  | high ripple | (d=.06; p=.08) | high ripple | (d=.55; p=.13) |
| Visual - Dorsal Attention | **Insufficient coverage** | | | |

**Table S5. Phase connectivity results of awakening from REM.** Reported here are phase locking value (PLV) results for all bands which displayed a significant difference between the awakening process to the wakefulness or sleep reference distributions (wRD and sRD respectively). The results are presented as the time in seconds from the intracranial awakening (IA) when compared to the RDs. Times are given for the convergence to the wRD and the divergence from the sRD. The difference between the awakening process and the RD prior to convergence or after the divergence was tested, on the channel level for all channels-pairs within a network or between two different networks, using a paired Wilcoxon test and assessed with Cliff’s d. The magnitude of the difference between the awakening process to the RDs is reported as relative deviation, which is the change in percentage compared to the RDs. The direction of change is represented as ↑↓ if the time after the awakening corresponded to an increase ↑ or decrease ↓ when compared to the reference distribution. ND- never diverged from the RD in any frequency band. NC- never converged back to the RD. Note: non significant results are reported with the median effect size and p value throughout the duration. Two wakefulness baselines were utilized: one from the prior evening and one from the prior morning. Any differences between these baselines, presented in this order, are detailed in the Table.

**Table S6**

| **#** | **Age at the SEEG** | **Sex** | **Age at Sz onset** | **SOZ (SEEG)** | **MRI** | **AED (mg/day)** | **Sleep related epilepsy** |
| --- | --- | --- | --- | --- | --- | --- | --- |
| **1** | 37 | M | 20 | Left mesio-temporal | bilateral frontal Periventricular nodular heterotopia | Keppra (1500), Tegretol (800), Clobazam (10) | No |
| **2** | 46 | M | 36 | Left anterior temporal and anterior insula | no abnormality | Tegretol (2100) | No |
| **3** | 57 | F | 8 | Right insula | no abnormality | Clobazam (10), Lamotrigine (300), tegretol (400) | No |
| **4** | 34 | F | 18 | Left temporo-occipital | no abnormality | Lamictal (400), Tegretol (1000) Clobazam (20) | No |
| **5** | 38 | M | 8 | Left temporo-occipital | left posterior insula, left posterior temporal and left inferior parietal atrophy and gliosis | Phenytoin (350), Clobazam (40), Levetiracetam (1500) | No |
| **6** | 40 | M | 26 | Left & Right mesial and lateral temporal | right hippocampal atrophy | Clobazam (30), Levetiracetam (3000) | No |
| **7** | 25 | M | 5 | Left temporo-occipital | Bilateral mesial occipital uligyria | Trileptal (1800), Levetiracetam (1000), Lamotrigine (400), Zonisamide (200) | No |
| **8** | 29 | M | 21 | Left mesio-temporal | Left frontal polymicrogyria, Left mesio-temporal | Tegretol (1200), Clobazam (20) | No |
| **9** | 24 | M | 11 | Left & Right mesial and lateral temporal | R smaller hippocampus and Left malformed hippocampus | Lacosamide(400), Clobazam (15) | No |
| **10** | 30 | F | 20 | bilateral mesio-temporal | no abnormality | Clobazam (10), Trileptal (1800), Topiramate (25) | Yes (seizures often nocturnal) |
| **11** | 61 | F | 28 | Left mesio-temporal | Bilateral hippocampal atrophy (L>R) | Levetiracetam (1500), Lacosamide (400 mg) | No |
| **12** | 21 | F | 10 | R mesio-temporal | no abnormality | Lamictal (400) | No |
| **12** | 26 | F | 16 | Left mesio-temporal | no abnormality | Lamictal (550), Vimpat (500), Fycompa (8) | No |
| **14** | 51 | M | 30 | Left L insula | vascular lesion in the subcortical white matter of the left frontal lobe adjacent to the third frontal gyrus | Lacosamide (400), Tegretol (2000), Fycompa (12) | No |
| **15** | 32 | M | 17 | Left mesio-temporal | no abnormality | Tegretol (1000), Levetiracetam (3000), Clobazam (10) | No |
| **16** | 42 | F | 11 | R insula | no abnormality | Trileptal (2100), Clobazam (30) | Yes (mostly from sleep) |
| **17** | 46 | M | 33 | Left widespread posterior quadrant | Left parietal-occipital lesion | Lacosamide (400), Lamotrigine (400), Tegretol (800),  Perampanel (10) | No |
| **18** | 29 | M | 22 | R perisylvian | no abnormality | Phenobarbital (120), Levetiracetam (2000), Tegretol (1000) | No |

**Table S6. Patient characteristics.** Abbreviations: Sz- Seizure, SOZ- Squire onset zone, SEEG- Stereoelectroencephalography, MRI- Magnetic Resonance Imaging, ASM- anti-seizure medication, mg- milligram

**Table S7**

| Region | Band | Correlation |
| --- | --- | --- |
| Inferior parietal lobule | delta | r=0.21 [0.15-0.25], p=0.01 [0.002-0.04] |
|  | theta | r=0.13 [0.08-0.18], p=0.06 [0.02-0.10] |
|  | alpha | r=0.08 [0.03-0.12], p=0.23 [0.15-0.32] |
|  | beta | r=0.06 [0.02-0.09], p=0.35 [0.19-0.53] |
|  | low gamma | r=0.10 [0.04-0.13], p=0.30 [0.23-0.49] |
|  | high gamma | r=0.06 [0.03-0.09], p=0.43 [0.19-0.68] |
|  | low ripple | r=0.12 [0.06-0.15], p=0.53[0.08-0.96] |
|  | high ripple | r=0.09 [0.05-0.14], p=0.13 [0.02-0.35] |
| Central operculum and opercular part of inferior frontal gyrus | delta | r=0.15 [0.08-0.24], p=0.07 [0.03-0.17] |
|  | theta | r=0.13 [0.09-0.16], p=0.32 [0.20-0.42] |
|  | alpha | r=0.06 [0.02-0.16], p=0.76 [0.48-1] |
|  | beta | r=0.12 [0.08-0.19], p=0.23 [0.12-0.38] |
|  | low gamma | r=0.08 [0.03-0.10], p=0.27 [0.14-0.80] |
|  | high gamma | r=0.03 [0.01-0.09], p=0.86 [0.78-1] |
|  | low ripple | r=0.10 [0.02-0.15], p=0.53 [0.18-0.83] |
|  | high ripple | r=0.07 [0.03-0.14], p=0.17 [0.07-0.37] |
| Superior, middle, and orbital frontal gyri and anterior part of inferior frontal gyrus | delta | r=0.35 [0.15-0.41], p=0.005 [0.001-0.06] |
|  | Theta | r=0.15 [0.07-0.20], p=0.03 [0.007-0.08] |
|  | alpha | r=0.10 [0.05-0.15], p=0.19 [0.10-0.45] |
|  | beta | r=0.12 [0.02-0.19], p=0.38 [0.18-0.76] |
|  | low gamma | r=0.04 [0.01-0.10], p=0.52 [0.24-1] |
|  | high gamma | r=0.08 [0.02-0.12, p=0.34 [0.15-0.65] |
|  | low ripple | r=0.10 [0.01 -0.15], p=0.16 [0.08-0.77] |
|  | high ripple | r=0.09 [0.04-0.13], p=0.22 [0.07-0.58] |
| Insula | delta | r=0.11 [0.04-0.15], p=0.51 [0.27-0.94] |
|  | theta | r=0.06 [0.01-0.10], p=0.74 [0.40-1] |
|  | alpha | r=0.09 [0.05-0.14], p=0.85 [0.24-1] |
|  | beta | r=0.13 [0.03-0.21], p=0.49 [0.17-0.69] |
|  | low gamma | r=0.11 [0.03-0.14] , p=0.64 [0.20-0.81] |
|  | high gamma | r=0.05 [0.01-0.12], p=0.68 [0.27-1] |
|  | low ripple | r=0.09 [0.06-0.16], p=0.78 [0.13-1] |
|  | high ripple | r=0.05 [0.01-0.09], p=0.93 [0.87-1] |
| Superior parietal lobule | delta | r=0.24 [0.10-0.31] , p=0.004 [0.001-0.014] |
|  | theta | r=0.16 [0.08-0.20] , p=0.03 [0.003-0.09] |
|  | alpha | r=0.05 [0.02-0.13], p=0.21 [0.15-0.67] |
|  | beta | r=0.08 [0.04-0.11], p=0.39 [0.14-0.70] |
|  | low gamma | r=0.06 [0.01-0.10], p=0.09 [0.02-0.15] |
|  | high gamma | r=0.11 [0.02-0.16-, p=0.83 [0.14-1] |
|  | low ripple | r=0.02 [0.01-0.07], p=0.53 [0.19-1] |
|  | high ripple | r=0.04 [0.01-0.09], p=0.43 [0.25-1] |
| Middle and inferior temporal gyrus, temporal pole, and planum polare | delta | r=0.12 [0.06-0.17], p=0.02 [0.004-0.09] |
|  | theta | r=0.16 [0.05-0.20], p=0.12 [0.02-0.48] |
|  | alpha | r=0.05 [0.01-0.20], p=0.35 [0.08-0.75] |
|  | beta | r=0.09 [0.03-0.15], p=0.75 |
|  | low gamma | r=0.07 [0.02-0.11], p=0.95 [0.84-1] |
|  | high gamma | r=0.04 [0.01-0.13], p=0.73 [0.10-1 |
|  | low ripple | r=0.08 [0.04-0.15], p=0.51 [0.26-0.79] |
|  | high ripple | r=0.11 [0.05-0.17], p=0.76 [0.26-1] |

**Table S7. Correlation between power in scalp and intracranial EEG during awakening from NREM sleep.** This table presents the Pearson correlation coefficients for each frequency band, comparing the median power in each intracranial region to the power recorded in the frontal/central scalp EEG. The median and range of the correlation coefficients and the corresponding FDR corrected p-values, are reported across the available patients for each region.

**Table S8**

| Region | Band | Correlation |
| --- | --- | --- |
| Anterior and middle cingulate gyrus | delta | r=0.02 [0.01-0.04], p=0.85 [0.56-1] |
|  | theta | r=0.07 [0.04-0.10], p=0.76 [0.45-0.98] |
|  | alpha | r=0.04 [0.01-0.09], p=0.93 [0.80-1] |
|  | beta | r=0.08 [0.02-0.12], p=0.87 [0.76-1] |
|  | low gamma | r=0.02 [0.01-0.04], p=1 [0.94-1] |
|  | high gamma | r=0.04 [0.01-0.08], p=0.95 [0.89-1] |
|  | low ripple | r=0.04 [0.01-0.06], p=0.73 [0.58-0.95] |
|  | high ripple | r=0.09 [0.02-0.16], p=0.78 [0.63-1] |
| Central operculum and opercular part of inferior frontal gyrus | delta | r=0.08 [0.03-0.14], p=0.24 [0.16-0.54] |
|  | theta | r=0.06 [0.01-0.13], p=0.59 [0.29-0.90] |
|  | alpha | r=0.03 [0.01-0.8], p=0.53 [0.32-1] |
|  | beta | r=0.09 [0.04-0.12], p=0.86 [0.58-1] |
|  | low gamma | r=0.02 [0.01-0.05], p=1 [0.80-1] |
|  | high gamma | r=0.03 [0.01-0.05], p=1 [0.63-1] |
|  | low ripple | r=0.05 [0.01-0.12], p=0.77 [0.18-1] |
|  | high ripple | r=0.05 [0.02-0.10], p=0.64 [0.20-0.93] |
| Inferior parietal lobule | delta | r=0.10 [0.02-0.14], p=0.13 [0.05-0.24] |
|  | theta | r=0.03 [0.01-0.06], p=0.24 [0.13-0.57] |
|  | alpha | r=0.09 [0.04-0.12], p=0.63 [0.42-1] |
|  | beta | r=0.03 [0.01-0.08], p=0.38 [0.19-0.70] |
|  | low gamma | r=0.06 [0.01-0.10], p=0.75 [0.41-1] |
|  | high gamma | r=0.04 [0.01-0.09], p=0.43 [0.25-0.79] |
|  | low ripple | r=0.01 [0.01-0.02], p=1 [0.78-1] |
|  | high ripple | r=0.01 [0.01-0.02], p=1 [0.78-1] |
| Insula | delta | r=0.04 [0.01-0.11], p=0.73 [0.30-1] |
|  | theta | r=0.04 [0.01-0.15], p=1 [0.43-1] |
|  | alpha | r=0.03 [0.01-0.07], p=0.84 [0.26-1] |
|  | beta | r=0.07 [0.02-0.10], p=0.96 [0.91-1] |
|  | low gamma | r=0.02 [0.01-0.04], p=1 [0.72-1] |
|  | high gamma | r=0.03 [0.01-0.05], p=1 [0.88-1] |
|  | low ripple | r=0.07 [0.03-0.12], p=0.89 [0.64-1] |
|  | high ripple | r=0.03 [0.01-0.06], p=0.97 [0.85-1] |
| Medial and basal temporal region | delta | r=0.11 [0.03-0.19], p=0.18 [0.06-0.45] |
|  | theta | r=0.09 [0.04-0.14], p=0.26 [0.16-0.50] |
|  | alpha | r=0.02 [0.01-0.05], p=0.47 [0.29-0.85] |
|  | beta | r=0.03 [0.01-0.08], p=0.75 [0.50-1] |
|  | low gamma | r=0.07 [0.02-0.10], p=0.66 [0.42-0.90] |
|  | high gamma | r=0.03 [0.01-0.08], p=1 [0.65-1] |
|  | low ripple | r=0.08 [0.03-0.11], p=0.39 [0.15-0.60] |
|  | high ripple | r=0.06 [0.02-0.10], p=0.52 [0.30-0.79] |
| Medial frontal cortex | delta | r=0.13 [0.05-0.18], p=0.13 [0.07-0.32] |
|  | theta | r=0.03 [0.01-0.05], p=0.36 [0.20-0.49] |
|  | alpha | r=0.07 [0.01-0.13], p=0.33 [0.14-0.64] |
|  | beta | r=0.03 [0.01-0.07], p=0.71 [0.54-1] |
|  | low gamma | r=0.04 [0.01-0.08], p=1 [0.80-1] |
|  | high gamma | r=0.03 [0.01-0.05, p=1 [0.94-1] |
|  | low ripple | r=0.02 [0.01-0.09], p=1 [0.69-1] |
|  | high ripple | r=0.06 [0.02-0.07], p=0.82 [0.68-1] |
| Middle and inferior temporal gyrus, temporal pole, and planum polare | delta | r=0.06 [0.01-0.12], p=0.56 [0.25-0.96] |
|  | theta | r=0.03 [0.01-0.06], p=0.53 [0.23-0.97] |
|  | alpha | r=0.05 [0.02-0.12], p=0.45 [0.15-0.85] |
|  | beta | r=0.05 [0.01-0.10], p=0.86 [0.56-1] |
|  | low gamma | r=0.02 [0.01-0.07], p=1 [0.42-0.96] |
|  | high gamma | r=0.01 [0.01-0.02], p=1 [0.78-1] |
|  | low ripple | r=0.04 [0.01-0.09], p=0.88 [0.67-1] |
|  | high ripple | r=0.06 [0.02-0.14], p=0.76 [0.58-1] |
| Superior, middle, and orbital frontal gyri and anterior part of inferior frontal gyrus | delta | r=0.03 [0.01-0.05], p=0.09 [0.03-0.24] |
|  | theta | r=0.03 [0.01-0.08], p=0.28 [0.15-0.45] |
|  | alpha | r=0.08 [0.03-0.13], p=0.36 [0.20-0.64] |
|  | beta | r=0.04 [0.01-0.06], p=0.53 [0.46-0.84] |
|  | low gamma | r=0.06 [0.02-0.15], p=0.72 [0.52-1] |
|  | high gamma | r=0.05 [0.01-0.12], p=0.83 [0.67-1] |
|  | low ripple | r=0.02 [0.01-0.05], p=1 [0.92-1] |
|  | high ripple | r=0.02 [0.01-0.10], p=1 [0.86-1] |
| Superior temporal gyrus | delta | r=0.04 [0.02-0.07], p=0.32 [0.12-0.75] |
|  | theta | r=0.03 [0.01-0.05], p=0.72 [0.54-0.96] |
|  | alpha | r=0.02 [0.01-0.08], p=0.21 [0.13-0.64] |
|  | beta | r=0.05 [0.03-0.13], p=0.63 [0.25-0.88] |
|  | low gamma | r=0.01 [0.01-0.04], p=0.42 [0.18-0.73] |
|  | high gamma | r=0.03 [0.01-0.09], p=0.86 [0.49-1] |
|  | low ripple | r=0.04 [0.01-0.13], p=0.65 [0.31-1] |
|  | high ripple | r=0.01 [0.01-0.02], p=1 [0.74-1] |
| Medial parietal lobe | delta | r=0.07 [0.03-0.10], p=0.21 [0.10-0.74] |
|  | theta | r=0.03 [0.01-0.08], p=0.53 [0.24-0.93] |
|  | alpha | r=0.08 [0.04-0.14], p=0.43 [0.25-0.83] |
|  | beta | r=0.02 [0.01-0.02], p=0.84 [0.60-1] |
|  | low gamma | r=0.02 [0.01-0.03], p=0.99 [0.85-1] |
|  | high gamma | r=0.06 [0.02-0.10], p=1 [0.92-1] |
|  | low ripple | r=0.03 [0.01-0.07], p=1 [0.83-1] |
|  | high ripple | r=0.04 [0.01-0.12], p=1 [0.89-1] |
| Superior parietal lobule | delta | r=0.03 [0.01-0.05, p=0.54 [0.37-0.78] |
|  | theta | r=0.04 [0.01-0.10], p=0.83 [0.48-1] |
|  | alpha | r=0.08 [0.03-0.11], p=0.72 [0.43-0.97] |
|  | beta | r=0.02 [0.01-0.04], p=0.79 [0.63-1] |
|  | low gamma | r=0.06 [0.02-0.09], p=0.56 [0.22-0.94] |
|  | high gamma | r=0.06 [0.01-0.14], p=0.73 [0.17-1] |
|  | low ripple | r=0.02 [0.02-0.05], p=0.36 [0.20-0.90] |
|  | high ripple | r=0.05 [0.03-0.10], p=0.26 [0.08-1] |
| Medial frontal cortex (including medial segment of superior frontal gyrus) | delta | r=0.05 [0.01-0.07], p=0.51 [0.30-0.86] |
|  | theta | r=0.12 [0.05-0.18], p=0.36 [0.18-0.70] |
|  | alpha | r=0.02 [0.01-0.06], p=0.73 [0.47-1] |
|  | beta | r=0.01 [0.01-0.02], p=0.49 [0.32-1] |
|  | low gamma | r=0.03 [0.01-0.06], p=0.86 [0.66-1] |
|  | high gamma | r=0.07 [0.02-0.13], p=0.67 [0.40-0.88] |
|  | low ripple | r=0.01 [0.01-0.02], p=0.96 [0.87-1] |
|  | high ripple | r=0.05 [0.01-0.10], p=0.91 [0.78-1] |
| Frontal operculum | delta | r=0.10 [0.04-0.17], p=0.58 [0.46-1] |
|  | theta | r=0.08 [0.04-0.15], p=0.73 [0.50-1] |
|  | alpha | r=0.02 [0.01-0.05], p=0.94 [0.86-1] |
|  | beta | r=0.06 [0.02-0.10], p=0.87 [0.34-1] |
|  | low gamma | r=0.02 [0.01-0.04], p=0.83 [0.58-1] |
|  | high gamma | r=0.02 [0.01-0.02], p=1 [0.69-1] |
|  | low ripple | r=0.03 [0.01-0.05], p=1 [0.88-1] |
|  | high ripple | r=0.03 [0.01-0.08], p=1 [0.91-1] |
| Transverse temporal gyrus and planum temporale | delta | r=0.03 [0.01-0.04], p=0.48 [0.19-0.69] |
|  | theta | r=0.01 [0.01-0.02], p=0.63 [0.28-0.95] |
|  | alpha | r=0.01 [0.01-0.02], p=0.93 [0.74-1] |
|  | beta | r=0.06 [0.02-0.10], p=0.68 [0.41-0.80] |
|  | low gamma | r=0.06 [0.01-0.09], p=1 [0.87-1] |
|  | high gamma | r=0.03 [0.01-0.08], p=1 [0.76-1] |
|  | low ripple | r=0.08 [0.05-0.12], p=0.92 [0.69-1] |
|  | high ripple | r=0.04 [0.01-0.07], p=0.79 [0.52-0.99] |

**Table S8. Correlation between power in scalp and intracranial EEG during awakening from REM sleep.** This table presents the Pearson correlation coefficients for each frequency band, comparing the median power in each intracranial region to the power recorded in the frontal/central scalp EEG. The median and range of the correlation coefficients and the corresponding FDR corrected p-values, are reported across the available patients for each region
